# Winery By-Products as Sustainable Functional Resources: An Overview of Composition, Green Extraction Technologies, and Industrial Valorization

**DOI:** 10.3390/molecules31193462

**Published:** 2026-09-28

**Authors:** Vanessa Hobeika, Nancy Zgheib, Nancy Nehme, Patricia Taillandier, Samar Azzi-Achkouty, Youssef El Rayess

**Affiliations:** 1 Department of Agriculture and Food Engineering, School of Engineering, Holy Spirit University of Kaslik, Jounieh P.O. Box 446, Lebanon; vanessa.l.hobeika@net.usek.edu.lb; 2 Department of Chemical Engineering, School of Engineering, Holy Spirit University of Kaslik, Jounieh P.O. Box 446, Lebanon; nancyzgheib@usek.edu.lb; 3 Processing and Biosystems Multidisciplinary Research Group, Holy Spirit University of Kaslik, Jounieh P.O. Box 446, Lebanon; 4 Faculty of Agricultural Engineering and Veterinary Medicine, Lebanese University, Dekwaneh P.O. Box 6573, Lebanon; nancy.nehme@ul.edu.lb; 5 Laboratoire de Génie Chimique, Université de Toulouse, CNRS. INPT. UT., F-31062 Toulouse, France; patricia.taillandier@toulouse-inp.fr

**Keywords:** winery by-products, polyphenols, green extraction, circular bioeconomy, functional foods, valorization

## Abstract

The wine industry generates substantial quantities of by-products, including grape pomace, seeds, skins, stems, wine lees, and wastewater, which are valuable sources of polyphenols, dietary fibers, lipids, proteins, and other bioactive compounds. This review examines the composition, extraction, and valorization of winery residues within a circular bioeconomy framework, with particular emphasis on the distribution and recovery of major polyphenols, including flavonoids, phenolic acids, stilbenes, anthocyanins, and tannins. Conventional extraction methods are critically compared with emerging green technologies, including ultrasound- and microwave-assisted extraction, pulsed electric fields, high-voltage electrical discharges, supercritical fluid extraction, and pressurized liquid extraction, considering their efficiency, sustainability, scalability, and industrial potential. The valorization of winery-derived ingredients in functional foods, edible packaging, nutraceuticals, cosmetics, animal feed, biofuels, and other value-added products is also discussed. Despite significant progress, raw material variability, process standardization, techno-economic feasibility, regulatory compliance, and consumer acceptance remain major barriers to industrial implementation. Integrating green extraction technologies with biorefinery approaches, life-cycle assessment, and process optimization could enhance resource efficiency and product quality, supporting the transition toward a more sustainable and circular wine industry.

## 1. Introduction

Grapes are among the most widely cultivated fruit crops worldwide, with a significant proportion of production dedicated to winemaking. According to OIV statistics, global wine production in 2025 is estimated to be between 228 and 235 million hectoliters [1]. While the wine industry contributes substantially to rural economies and cultural heritage, it simultaneously generates large volumes of organic by-products and residues. It is estimated that approximately 0.3–0.5 kg of by-products is generated per liter of wine produced, mainly in the form of grape pomace, seeds, and stems, with additional residues, such as wine lees and wastewater, formed during fermentation and clarification processes [2,3].

Traditionally, these by-products have been disposed of through landfilling, composting, or low-value applications, practices that raise environmental concerns due to high organic load, phytotoxicity, and greenhouse gas emissions [4]. In recent years, increasing regulatory pressure, coupled with the principles of sustainability and circular economy, has driven renewed interest in the valorization of winery by-products [5].

Sustainability in the wine industry involves the adoption of practices aimed at reducing environmental impacts while enhancing resource efficiency throughout the production chain, like precision agriculture and biological control strategies [6]. Beyond vineyard management, waste generation represents a major sustainability challenge in the wine industry. The recovery of polyphenols, dietary fibers, and other bioactive compounds from these by-products enables the development of value-added ingredients for agricultural, food, cosmetic, and pharmaceutical applications, in line with circular economy principles that emphasize waste reduction and efficient resource utilization [7].

Despite their promising composition, the effective valorization of grape and winery by-products remains technically and economically challenging. Variability in raw material composition, instability of bioactive compounds, and the need for efficient, scalable, and environmentally friendly extraction technologies pose significant obstacles to industrial implementation. Furthermore, the successful incorporation of by-product-derived ingredients into food and feed systems requires careful consideration of technological functionality, safety, sensory impact, regulatory compliance, and consumer acceptance. Addressing these challenges necessitates an integrated and multidisciplinary approach that combines chemical characterization, process optimization, application-driven research, and sustainability assessment.

The aim of this review is to provide a comprehensive and critical synthesis of current knowledge on the valorization of grape and winery by-products. Specifically, the review focuses on (i) the classification and chemical composition of major winery by-products, (ii) conventional and emerging extraction techniques for the recovery of polyphenols and other valuable compounds, and (iii) key application pathways, including their use as food additives, functional ingredients, biofuels, animal feed components, and other value-added products. Challenges and future research directions are also discussed.

## 2. Grape By-Product Composition

### 2.1. Classification and Chemical Composition of Winery By-Products

Winery by-products or grape by-products comprise a diverse group of solid, semi-solid, and liquid residues generated at different stages of the winemaking process, including grape pomace, seeds, skins, stems, wine lees, and winery wastewater (Table 1). Among these, grape pomace or grape marc is the most abundant solid by-product, representing approximately 20–30% of the processed grape mass, and consists mainly of skins, seeds, and residual pulp [8]. Its chemical composition is dominated by high levels of dietary fiber, both insoluble (cellulose, hemicellulose, and lignin) and soluble fractions (pectin), which may account for more than half of its dry matter, particularly in red grape varieties [9]. Grape skins are especially rich in polyphenolic compounds, such as anthocyanins, flavonols, flavan-3-ols, and condensed tannins, as well as polysaccharides and organic acids, making them a valuable source of natural antioxidants and colorants [10,11]. Grape seeds exhibit a distinct compositional profile characterized by a significant lipid fraction (typically 8–20% oil) and are rich in unsaturated fatty acids, mainly linoleic and oleic acids, together with tocopherols, phytosterols, proteins and high concentrations of flavan-3-ols and proanthocyanidins associated with strong antioxidant activity [12,13,14]. Grape stems, although often discarded, are primarily composed of lignocellulosic materials, including cellulose, hemicellulose, and lignin, and contain appreciable amounts of minerals and phenolic acids, though their polyphenolic diversity and extractability are generally lower than those of skins and seeds [15,16]. Wine lees, which are formed during alcoholic fermentation and subsequent clarification, largely consist of yeast biomass, tartrate salts, proteins, polysaccharides, minerals, and phenolic compounds adsorbed onto yeast cell walls, representing a secondary but increasingly recognized source of bioactive molecules [17,18]. In addition, winery wastewater contains dissolved organic matter, residual sugars, acids, and phenolics, contributing to its high chemical oxygen demand [19,20]. Overall, the chemical composition of winery by-products is highly heterogeneous and strongly influenced by grape variety, terroir, climatic conditions, viticultural practices, vinification techniques, and post-processing treatments, all of which affect the concentration, stability, and recovery potential of valuable compounds and must be carefully considered when designing sustainable and efficient valorization strategies.

### 2.2. Polyphenols: Chemistry and Structure

**Figure 1:**
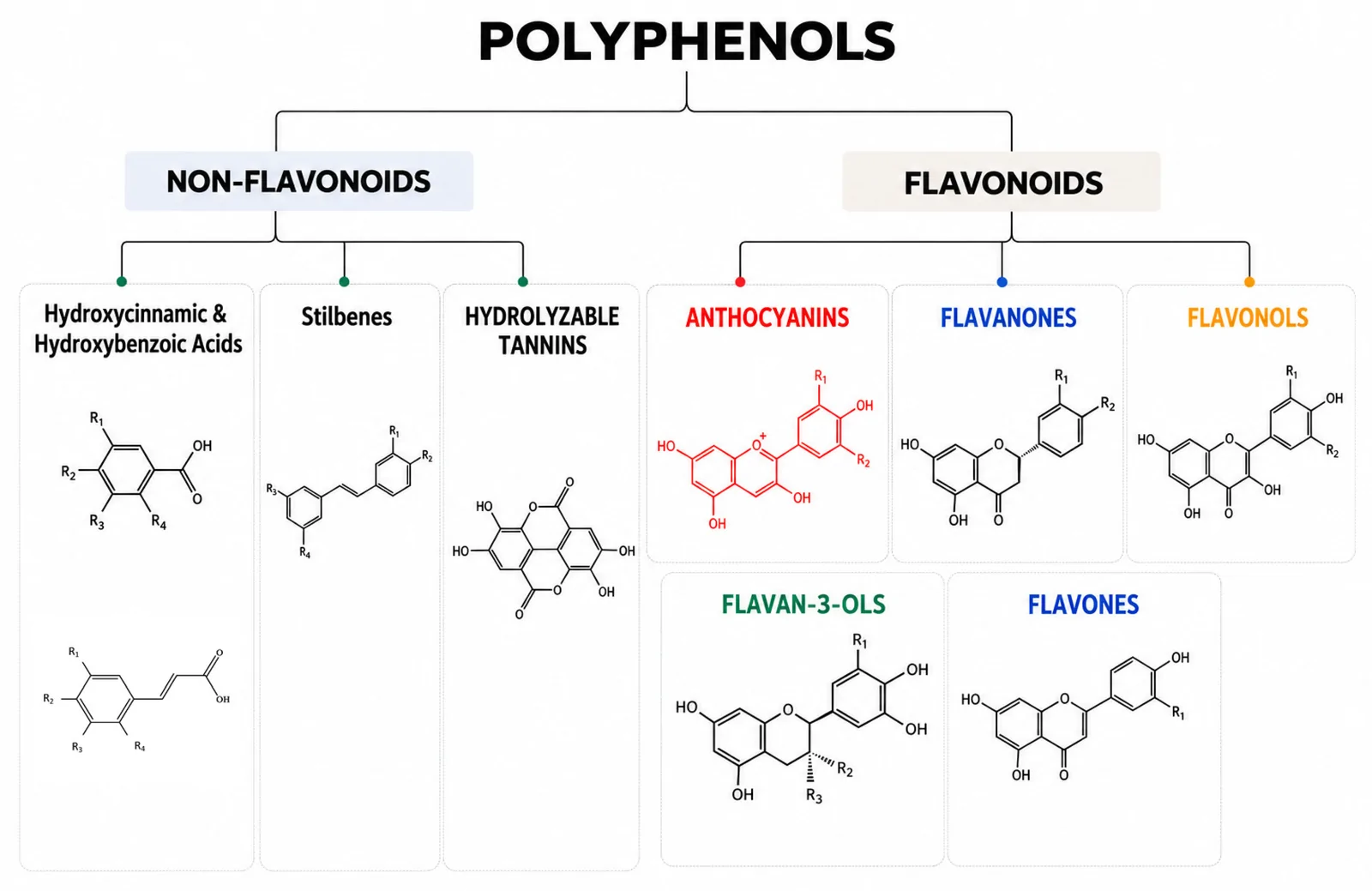
Classification of polyphenols and their sub-classes.

Grapes are one of the fruits containing the greatest content of phenolic substances. Polyphenols represent the most extensively studied and commercially valuable components of grape by-products, contributing significantly to their biological activities and potential applications across multiple industries. Polyphenols, as a group of phytochemicals, are divided into two main families: non-flavonoids and flavonoids [21]. Phenolics are responsible for some organoleptic properties, aroma, color, flavor, bitterness and astringency, and are thus known as sensory compounds [22]. The most abundant polyphenols in wines are phenolic acids, flavonoids, and tannins, as shown in Figure 1.

#### 2.2.1. Non-Flavonoids

Non-flavonoid polyphenols are a diverse group of compounds present in wine and its by-products, including hydroxybenzoic acids, hydroxycinnamic acids, stilbenes and hydrolyzable tannins. These compounds, though often overshadowed by flavonoids, play crucial roles in wine chemistry, sensory attributes, and health-promoting bioactivities. Their occurrence in grape skins, stems, pomace, and wine lees underscores their relevance not only to wine quality but also to the sustainable valorization of winemaking residues for food, pharmaceutical, and cosmetic industries [2,23].

Phenolic acids constitute a fundamental class of polyphenols in grape by-products, subdivided into hydroxybenzoic acids and hydroxycinnamic acids that may occur as free or conjugated.

Hydroxybenzoic acids—specifically gallic, protocatechuic, vanillic, and syringic acids—are predominantly distributed in grape seeds and pomace, confirming these by-products as principal reservoirs of this class of phenolic compounds [24]. Gallic acid is consistently identified as the predominant phenolic acid, occurring at concentrations of approximately 10–13 mg per 100 g dry weight, with levels varying according to grape variety and extraction parameters [25]. This compound serves as a precursor for hydrolyzable tannins and exhibits potent antioxidant and antimicrobial activities [22].

The most representative types of hydroxycinnamic acids are ρ-cinnamic, caffeic, ferulic, and sinapic acids. These compounds are partly responsible for wine browning and are the precursors of volatile phenolic compounds [22,23].

As for their localization in grapes, trans-caftaric and trans-fertaric acids are found in the pulp and are therefore readily extracted during the winemaking process. Trans and cis isomers of ρ-coutaric acid are less extractable due to their presence in the skin, and they are partially responsible for wine astringency [22].

The stilbene fraction identified in grapes and wines is dominated by trans-resveratrol and its derivatives, which are predominantly localized in the grape skin [26]. These compounds are synthesized as phytoalexins in response to biotic and abiotic stress, resulting in highly variable concentrations that depend on cultivars, environmental conditions, and agronomic practices. Resveratrol has been described by several authors as a stress-related metabolite, potentially functioning as a phytotoxic response to fungal infection or other biotic challenges. During winemaking, extended maturation and maceration periods are generally associated with increased resveratrol levels in the final product.

Grape pomace typically contains 0.3–2 mg of trans-resveratrol per 100 g dry weight, while oligomeric stilbenes, such as ε-viniferin and δ-viniferin, are also detected at lower concentrations [25]. In addition to resveratrol, wines and grape by-products may contain a diverse range of stilbenes, including piceid, piceatannol and glycosylated derivatives (e.g., astringin) [22].

Notably, grape stems exhibit particularly high stilbene concentrations, often containing two- to five-fold higher resveratrol levels than pomace [27]. This pronounced enrichment positions grape stems as a preferred raw material for stilbene recovery, especially for cosmetic and nutraceutical applications, where the anti-aging, antioxidant, and cardioprotective properties of resveratrol are highly sought after.

Hydrolyzable tannins are hydrolyzed by enzymatic, non-enzymatic, and pH changes into phenolic acids and sugars. The basic acids are gallic and ellagic acids; thus, tannins are classified as gallotannins and ellagitannins, each hydrolyzed in respective acids [28].

#### 2.2.2. Flavonoids

Flavonoids are diverse compounds differentiated by the oxidation degree of their central pyran ring, classified into flavones, flavonols, flavanones, flavononols, flavanols and anthocyanins [22]. They are crucial for the sensorial characteristics of wine and are found in pomace since they are present in the skin and seeds [29].

Luteolin is the only flavone found in wine, and as for isoflavones, genistein is the only one found. Flavones are known for their chemotaxonomic and pharmacological properties [22]. The main flavonones found in grapes and wine are eriodyctiol, naringenin, and hesperetin [22].

Flavan-3-ols, more specifically catechin and epicatechin, are mainly found in skins and seeds of grapes [26]. These monomeric constituents act as fundamental precursors for proanthocyanidins (condensed tannins), which comprise oligomeric and polymeric derivatives of flavan-3-ols. Fermented grape pomace seeds, particularly from cultivars such as Syrah and Alicante, exhibit remarkably high levels of flavan-3-ol monomers and procyanidins, accounting for up to 50% of the total phenolic content of grape pomace and contributing substantially to its overall bioactivity [30,31].

Flavonols are named 3-hydroxyflavones due to the presence of a hydroxyl group in the C3 position. Almost 90% of the flavonols found are 3,5,7-trihydroxylated derivatives [22]. Quercetin represents one of the predominant flavonol in most grape varieties, with concentrations in pomace extracts ranging from 2.17 to 2.85 mg/100 g [25]. Their presence in higher concentrations in pomace from rosé wine production compared to fully fermented red wines highlights the impact of maceration time on flavonol retention [32]. They are considered to be co-pigments of anthocyanins where they stabilize their color by altering their intensity [29].

Anthocyanins are produced during ripening of grapes [29]. Anthocyanins are exclusively present in the grape’s skin, and their extraction amount depends on conditions such as temperature and degree of disruption of grapes as well as the duration of the process [33,34]. Agents such as temperature, light, pH, oxygen, and others can chemically alter anthocyanins [29]. Despite extensive extraction during vinification, red grape pomace retains substantial quantities of anthocyanins, with total concentrations of approximately 67 mg of cyanidin-3-glucoside equivalents per 100 g dry weight, confirming its relevance as a residual yet valuable source of these pigments [35]. The anthocyanin profile of grape pomace is dominated by 3-O-glucosides of delphinidin, petunidin, and malvidin (bluish-red pigments), along with cyanidin (orange-red pigment) and peonidin (red pigment) [36]. Among these, malvidin-3-O-glucoside typically predominates, accounting for approximately 40–60% of total anthocyanins in most red grape varieties [24]. In addition, acylated anthocyanins, including acetylated, coumaroylated, and caffeoylated derivatives, are commonly detected and generally display enhanced chemical stability relative to their non-acylated counterparts [36]. Anthocyanin retention in grape pomace is strongly influenced by vinification practices. Pomace derived from rosé wine production, characterized by short maceration times (12–24 h), retains significantly higher anthocyanin levels than pomace from conventional red vinification involving prolonged maceration [32]. This variability highlights the potential for process-dependent, selective valorization strategies targeting anthocyanin-rich by-products.

Tannins are known for their astringent properties and for their ability to precipitate proteins [22]. Tannins are hydrophilic polymeric phenolics with a molecular weight ranging from 500 Da up to 30 kDa [37]. Tannins possess a polymeric structure rich in hydroxyl and other functional groups, often present as ester or hetero-ester linkages, which confer a strong binding affinity toward a wide range of molecules, particularly metals and proteins [28]. Tannins occur in all grapevine by-products, namely skins, seeds, and stems, but their concentration and structural composition differ markedly between these tissues.

Condensed tannins or proanthocyanins are usually present in the solid parts of grapes and in the pulp. Their location in grapes determines their quantity, structure, and degree of polymerization. The predominant condensed tannins in grapes are procyanidins and prodelphinidins, which are oligomeric and polymeric flavan-3-ols. Under strongly acidic conditions, these compounds can undergo depolymerization and conversion to the corresponding anthocyanidins, including cyanidin and delphinidin. Sensory characteristics of wine, such as color, taste, astringency, and bitterness, are mostly the responsibility of proanthocyanins [22]. These compounds have oxidation, condensation, and polymerization properties, leading them to have an important role in the wine aging process.

## 3. Extraction of Phenolic Compounds

Extraction methodologies exert a crucial influence on the recovery efficiency of polyphenolic compounds from grape pomace by modulating the processing time, solvent accessibility, and the structural integrity of the plant cell matrix. The effectiveness of a given method is largely dependent on its capacity to solubilize free phenolic compounds and to disrupt cell wall-bound fractions, which are chemically associated with structural polymers such as lignin and cellulose [38].

Research and studies are still ongoing to determine the most efficient and rapid method for the extraction of polyphenols [39]. Conventional solid–liquid extraction using solvents such as water, ethanol, and methanol remains one of the most widely used approach for polyphenol recovery due to its operational simplicity, accessibility, and applicability at both laboratory and industrial scales.

Traditional extraction methods can be time-consuming, energy-intensive, and associated with high operational costs and the use of environmentally undesirable solvents [40]. Limitations of traditional methods have led scientists to explore more ecofriendly and innovative techniques. Nonconventional methods are being applied to maximize profitability and decrease costs, such as supercritical fluid extraction, subcritical water extraction–pulsed electrical fields, ultrasound-assisted extraction, microwave-assisted extraction, accelerated solvent extraction [41], and the use of hydrotropic solvents [31,41,42]. The nature and concentration of extracted polyphenols are affected by different elements, such as grape variety and maturity and the conditions of the winemaking process (temperature, use of enzymes, frequency of pump-over…) [34].

### 3.1. Conventional Techniques

#### 3.1.1. Solid–Liquid Extraction (SLE)

Conventional solid–liquid extraction (SLE) involves macerating grape pomace with an appropriate solvent to promote the solubilization and transfer of phenolic compounds from the solid matrix into the liquid phase. Soxhlet extraction is another conventional solvent-based technique, in which the solid matrix makes repeated contact with a freshly condensed solvent through successive reflux cycles. Since polyphenols are of polar nature, the solvents used should be polar too (such as ethanol and methanol). Moutinho et al. [36] studied the extraction of polyphenols from various red grape cultivars using different solvents. The results show that optimal extraction was attained using ethanol 70% with HCl 3.5% at 60 °C, yielding a total phenolic content of 44.93 mg of gallic acid equivalent (GAE)/g dry weight (DW) and 22.95 mg of catechin equivalent (CE)/g DW as the total flavonoid content, whereas other researchers used acidified acetone–methanol mixtures, and they recovered lower phenolic contents, around 2.3 mg GAE/g DW [43].

Various factors can affect the extraction process, such as the solid–liquid ratio, temperature, time, pH, particle size, and solvent used [44]. The extraction process becomes more effective when the temperature increases because this allows for better permeability of cell walls and increased extraction of phenolic compounds, which results in improved heat and mass transfer through plant materials [45]. Although the extraction process benefits from higher temperatures, resulting in better yields in less time, an increased temperature has limits that cannot be exceeded [46]. Excessively high temperatures or prolonged thermal exposure may promote the degradation or transformation of thermolabile phenolic compounds. The optimal extraction temperature therefore depends on the target phenolic compounds, extraction medium, treatment duration, and characteristics of the plant matrix.

Oreopoulou et al. [45] demonstrated that the extraction process becomes faster when the solvent–solid ratio increases because this creates a larger concentration difference between the solid material and the liquid solvent, but the polyphenol extracts will be lower with many impurities. The efficiency of the extraction process depends on the size of the particles used in the extraction process. Smaller particles create more surface area, which allows for better solvent contact to occur, resulting in higher mass transfer rates, which leads to more effective extraction processes with less time [47]. Thus, grinding the raw material is an important pretreatment to ensure better results [48]. However, pre-extraction processes, such as drying and grinding, can cause the denaturation of some important compounds [40]. SLE offers an affordable and straightforward method which accurately extracts phenolic compounds from grapevine plants without requiring costly machinery. This method is the most widespread method used at the laboratory and industrial scale, but it consumes a lot of energy and time [49]. The implementation of this method, which uses shakers or magnetic stirrers, enables researchers to achieve their desired sample throughput. The technique proves to be highly effective for normal testing of phenolic compounds and commercial extraction processes which use ethanol as the solvent generally recognized as safe (GRAS) [47]. Table 2 summarizes different extractions of polyphenols from grape pomace given in the literature.

#### 3.1.2. Hot Pressurized Liquid Extraction (HPLE)

HPLE consistently outperforms conventional atmospheric solid–liquid extraction among traditional extraction techniques. Operating under subcritical conditions (≈10 MPa and 90–150 °C), HPLE markedly enhances solvent penetration and mass transfer, resulting in significantly higher extraction yields within substantially reduced processing times. In contrast, atmospheric solid–liquid extraction typically requires extraction periods exceeding one hour, increasing the risk of oxidative degradation and consequent losses of phenolic compounds [58,59]. Quantitative assessments indicate that HPLE improves the recovery of major polyphenolic families, including flavanols (17–89%), phenolic acids (17–1000%), and flavonols (81–258%), relative to atmospheric extraction methods [60]. These enhancements are supported by scanning electron microscopy observations, which reveal a pressure-induced collapse of the plant matrix and rupture of intracellular vacuoles, thereby facilitating the release of entrapped bioactive compounds [61].

### 3.2. Alternative Green Techniques

In recent years, several alternative extraction technologies have been proposed and tested to replace conventional solvent extraction methods in order to improve economic efficiency and environmental sustainability. These emerging techniques aim to significantly reduce solvent consumption while enhancing extraction kinetics and decreasing energy demand [62].

**Figure 2:**
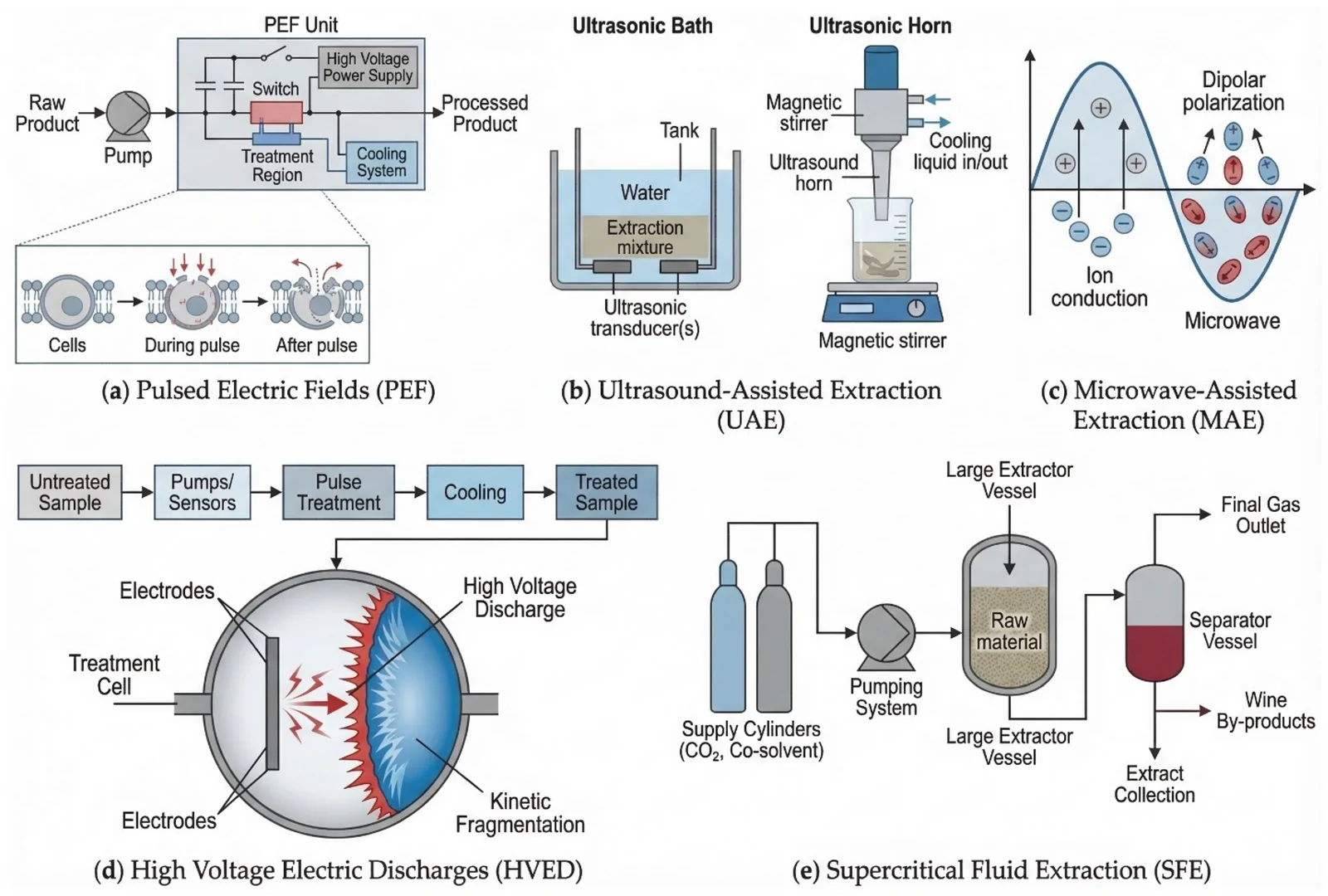
Principles of alternative extraction processes.

As illustrated in Figure 2a–e, these techniques rely on physical mechanisms that enhance cell disruption and mass transfer while reducing solvent and energy consumption.

#### 3.2.1. Pulsed Electric Fields (PEFs)

The pulsed electric field technique operates on the principle of electroporation, whereby the application of a pulsed external electric field induces the formation of pores in the cell membrane (Figure 2a). During treatment, the electric field (E ≠ 0) causes the accumulation of electrical charges on both sides of the cell membrane, leading to membrane polarization and the generation of a transmembrane potential (Vm). When this potential exceeds the dielectric strength of the membrane, membrane permeability increases significantly, and pores are formed [63]. Depending on the intensity and duration of the treatment, these pores may be reversible or irreversible. A typical PEF system consists of three main components: a high-voltage pulse generator, a treatment chamber where the sample is exposed to the electric field, and a control unit used to monitor and regulate the process parameters [64]. This technique requires submersion in water in order to improve electrical contact between electrodes [44], as well as a high electric field strength ranging from a few to several tens of kV/cm. The efficiency of pulsed electric field extraction depends on several factors related to both the raw material and the processing conditions. These include the location and size of the target compounds within plant cells, as well as solvent characteristics and sample properties, such as shape, size, pH, and electrical conductivity. Among the operational parameters, electric field strength plays a crucial role, as it affects the physicochemical properties of the extracted molecules, including diffusivity, solubility, viscosity, and surface tension. In PEF systems, by-products are exposed to short high-voltage pulses (typically 10–80 kV/cm) lasting from microseconds to milliseconds, which induce membrane permeabilization and facilitate compound release. Extraction performance can be adjusted by modifying parameters such as pulse frequency, pulse width, pulse shape, electric field intensity, and treatment time, the latter being influenced by the flow rate and the volume inside the treatment chamber. Although the PEF method is considered a non-thermal technique typically applied at ambient temperature, excessive treatment duration may increase product temperature, potentially affecting extraction efficiency [65,66,67].

Under continuous PEF conditions (25 kV, 10 Hz, and a diameter of chamber of 25 mm), Medina-Meza and Barbosa-Cánovas [68] showed that PEF-induced electroporation significantly increased extraction yields several folds compared to hot-water extraction while maintaining low temperature rises, thereby limiting thermal degradation of sensitive compounds. Brianceau et al. showed that PEF treatments increased the total polyphenol recovery but modified the composition, notably increasing the anthocyanin-to-flavanol ratio [69]. They demonstrated that PEFs could reduce solvent consumption and extraction times (reduction of up to 100 min). Under optimized pulsed electric field (PEF) conditions of 4.60 kV/cm and 20 kJ/kg, Carpentieri et al. demonstrated a marked enhancement in the recovery of bioactive compounds from grape pomace [70]. Relative to untreated samples, the treatment resulted in 15%, 60%, 23%, and 42% increases in total phenolics, flavonoids, anthocyanins, and tannins, respectively. From an industrial perspective, the techno-economic analysis by Soltanipour et al. [71] showed that an optimized PEF-assisted process reduced the unit production cost by ~2.70% and increased the return on investment by over 8% while also enabling operation under milder extraction conditions (lower temperature, ethanol concentration, or time), reinforcing its sustainability advantages.

#### 3.2.2. High-Voltage Electrical Discharge (HVED)

HVED (Figure 2d) represents another non-thermal electrotechnology for the enhancement of polyphenol extraction from grape by-products. It is conceptually related to PEFs but differs fundamentally in the spatial localization of energy delivery. During the pre-breakdown phase, the process is initiated by the formation and propagation of an electrical streamer characterized by the development of thin ionized vapor channels. When the local electric field intensity is sufficiently high, the streamer propagates toward the counter electrode. Once the streamer bridges the gap and reaches the plate, the breakdown phase occurs, resulting in a rapid and intense electrical discharge [44,72,73]. The two phases are accompanied by several secondary physicochemical phenomena, such as the propagation of high-intensity pressure shock waves, the emission of ultraviolet (UV) radiation, cavitation due to rapid gas bubble formation and collapse, and the generation of reactive species, such as radicals and ions [44,72,73]. HVED efficiency is influenced by several electrical, process and matrix parameters. These parameters include voltage, energy input, pulse frequency, duration, reactor design and electrode configuration, solvent composition, pH, temperature, solid–liquid ratio, matrix particle size and structure [73,74].

Barba et al. reported that treating Dunkelfelder grape pomace in a needle-plate system (40 kV, 10 kA; 10 μs pulses; 0.5 Hz; up to 218 kJ/kg) significantly enhanced total phenolic recovery compared to ultrasounds and PEF methods [75]. Similarly, Brianceau et al. optimized HVED treatment of grape stems (up to 188 kJ/kg), showing that acidic conditions (pH 2.50), a short treatment time (4 ms), and 50% ethanol improved phenolic extraction by ~35%, particularly increasing flavan-3-ols and flavonols [69]. Lončarić et al. [76] further demonstrated that in fungus-resistant grape pomace, optimal conditions (60 kV/cm, 100 Hz, 15 min, 50% ethanol) yielded 30.24 mg GAE/g DW with high antioxidant activity (2.17 mmol Trolox/g DM), even at a relatively low energy input (22.27 kJ/kg). Moreover, Deng et al. developed a continuous HVED system (12 kV, 30% ethanol, and 50 mL/min), achieving a polyphenol yield of 6.78% in only 6 min, with significantly lower energy consumption than conventional maceration [77].

#### 3.2.3. Microwave-Assisted Extraction (MAE)

MAE is one of the most studied green technologies for the recovery of polyphenols from grape by-products. The fundamental mechanism of MAE is based on electrical heating initiated by the rotational energy levels of particles using non-ionizing frequencies ranging between 300 MHz to 300 GHz. Radiation induces ionic conduction and dipole rotation mechanisms, leading to rapid internal heating cell wall disruption and enhanced solvent penetration [63]. This results in increased mass transfer and the improved release of intracellular phenolic compounds, particularly anthocyanins and proanthocyanidins [38,64]. Many factors influence MAE extraction efficiency, such as matrix nature, particle size, solvent type, solvent–material ratio, microwave power, application time and temperature [78,79].

Several studies have demonstrated the effectiveness of MAE under optimized operating conditions. For example, microwave treatment at high power (up to 1000 W for 10 min) using water acidified with 2% citric acid significantly increased the total phenolic content and antioxidant activity in grape pomace extracts [80]. The extraction of polyphenols from Chardonnay grape pomace using the MAE method was optimized by [81], finding that the optimal conditions are 10 min of extraction time, 48% ethanol and 1.77 g of solid mass. High antioxidant activity (87% DPPH inhibition) was also registered. A microwave–vacuum configuration operating at 80 W and 20 in Hg (vacuum) for 10 min significantly reduced the drying time and achieved a 64.5% recovery yield of bioactive compounds [82]. Liazid et al. demonstrated that the extraction solvent produced the greatest impact on MAE results in the extraction of anthocyanins from grape skins, while other factors (time, temperature…) had lower effects [83]. The combination of multiple solvents allows for different compounds to be selectively extracted through their various solvent properties [84]. Recently, the use of green solvents, such as natural deep eutectic solvents (NaDES), were used in MAE to further enhance extraction efficiency. For instance, MAE combined with choline chloride-based solvents enabled rapid extraction (≈3–4 min) with high proanthocyanidin yields (up to 135 mg/g of pomace), highlighting the synergy between microwave energy and green solvent systems [85]. Panic et al. demonstrated that the use of NaDES composed of choline chloride:citric acid in 30% water resulted in the highest anthocyanin recovery with simultaneous ultrasound/microwave-assisted extraction for 10 min [86].

Although MAE is considered a sustainable method with many advantages (lower energy and solvent consumption, and higher yields with greater quality of extracts), it is considered expensive due to the special equipment needed. Moreover, this method is mainly used with limited sample sizes due to the time needed for achieving experimental parameters, post-extraction cooling, and depressurizing the system in between samples.

#### 3.2.4. Ultrasound-Assisted Extractions (UAEs)

Ultrasounds are mechanical waves attributed to the cavitation process in a liquid medium with frequencies between 20 KHz to 10 MHz. This process is the result of the creation, the expansion and the implosion of microbubbles formed from gases initially dissolved in the liquid, leading to surface peeling, erosion and particle breakdown, allowing solvents to extract phenols easily in a short time [87,88]. UAE acts with combined mechanisms such as fragmentation, erosion, capillarity, detexturation and sonoporation [89]. Parameters influencing the efficiency of the UAE technique can be divided into three main categories: (i) ultrasound conditions: power, frequency amplitude and intensity; (ii) process conditions: shape and size of the reactors, solvent type, temperature, time, presence of dissolved gases and solid–liquid ratio; and (iii) matrix characteristics: particle size, cell wall composition and grape variety [90]. The selection of solvent type is extended in UAE treatments, allowing for the substitution of toxic organic solvents with GRAS.

UAE was shown to enhance the recovery of total phenolics, anthocyanins, tannins and antioxidant activity from grape pomace compared to traditional techniques. The optimized conditions were determined to be 60% ethanol with a pH of 2, a solvent–solid ratio of 50:1, and an amplitude of 100% for 5.05 min at a temperature of 60 °C [91]. Cordova et al. showed that extraction plateaus can be reached within 5 min, with prolonged sonication yielding only marginal improvements while increasing energy consumption [92]. The temperatures used during UAE treatments range between 25 and 60 °C. The optimal extraction temperature in ultrasound-assisted extraction systems depends on the solvent and targeted efficiency. In ethanol-based systems, temperatures around 35 °C are often considered optimal [93], while higher temperatures in the range of 50–60 °C are frequently applied to improve diffusion and mass transfer rates [92].

Recently, NaDES have emerged as promising green extraction media for ultrasound-assisted extraction of polyphenols, offering advantages in both efficiency and sustainability [94]. For instance, in optimized UAE conditions using a choline chloride–lactic acid–water NaDES system, the total polyphenol content reached 4.14 mg GAE/g DW, with flavonoids of around 15.80 mg CE/100 g DW and anthocyanins at 57.58 mg C3G/g DW, values comparable to ethanol systems but obtained under greener conditions [93]. Panic et al. showed that the highest concentration of total polyphenols was achieved when betaine/glucose with 30% water was used, and the extraction was conducted at an amplitude of 20%, for 8.50 min, and with a mass-to-volume ratio of 0.06 [86]. Moreover, NaDES significantly enhances bioactivity and functional performance. Extracts obtained using choline chloride-based NaDES showed improved antioxidant properties and antidiabetic activity compared to conventional solvents. In addition, NaDES systems improved polyphenol stability, preserving the anthocyanin structure during storage (e.g., 60 days at 4 °C) better than ethanol-based extracts [95].

The advantages of the UAE method are lower time and energy consumption, elevated penetration of the solvent resulting in greater yields, and lower solvent consumption [75]. Moreover, other studies found that UAE is effective for anthocyanin recovery since it protects thermolabile pigments more than other treatments that use elevated temperatures and toxic chemicals [38]. However, this treatment is mainly used for short extractions and a small number of samples since it must be done one batch at a time. During the use of the ultrasonic bath, water will be heated by the waves, necessitating some time for cooling between the runs.

#### 3.2.5. Supercritical Fluid Extraction (SFE)

A supercritical state is when a fluid is forced to a pressure and temperature above its critical point, conferring many properties to the fluid, such as gas-like low viscosity and high diffusivity [96]. A supercritical fluid can easily penetrate the matrix with a rapid mass transfer rate, offering advantages such as rapid extraction, low costs, little or no consumption of organic solvents, selective extraction and preservation of bioactive compounds [97]. Many parameters can influence the SFE process, such as solvent selection, pressure, temperature, solvent type and ratio, co-solvent use, time, flow rate, particle size and the moisture of the material [97,98]. One of the main solvents used in SFE is carbon dioxide (CO_2_), which has a relatively low critical temperature of 31.10 °C and a low critical pressure of 7.40 MPa. Supercritical CO_2_ properties facilitate mass transfer and yield to a solvent-free extract [44]. To extract polar compounds, a co-solvent, such as ethanol, is often added to CO_2_ to enhance the solubility of polar compounds, resulting in a higher yield of the targeted material [87].

Researchers investigated the effect of an ethanol–water mixture for phenolic recovery from grape marc, and the highest yields were obtained with 6 mL/min of CO_2_ and 10% ethanol/water [99]. The same authors showed that combining UAE and SFE for polyphenol extraction increased polyphenol yields compared to controls. Tuhanioglu et al. studied the optimization of supercritical CO_2_ extraction operating conditions on muscadine grape pomace using a response surface methodology [100]. The optimal conditions were identified to be 20 MPa, 60 °C, and 15% co-solvent, yielding high recoveries of total phenolics (≈2491 mg/100 g), flavonoids (≈674 mg/100 g), and resveratrol. They showed that higher temperatures improved solute diffusivity and solubility, while lower pressures enhanced mass transfer, indicating a balance between density-driven solubility and diffusional limitations. Da Porto and Natolino studied the implementation of sequential SFE processes, which allow for fractionation of compounds based on polarity [40]. The initial extraction step using pure SFE-CO_2_ (280 bar, 70 °C) selectively removes nonpolar compounds, such as waxes and policosanol. The second stage, using CO_2_ with a ~10% ethanol–water co-solvent (≈80 bar, 40 °C), achieved efficient extraction of polar phenolic compounds, yielding up to 21.60 mg GAE/g DW. Kinetic modeling (Broken and Intact Cell model) demonstrated that internal diffusion is the main rate-limiting step, which can be significantly reduced by co-solvent addition. In the same direction, a sequential three-stage biorefinery approach has been proposed by [101] to fractionate grape pomace components according to polarity, particularly in Isabella grape pomace. In the first stage, supercritical CO_2_ extraction (31.70 MPa, 58.90 °C) targeted lipophilic constituents, producing 6.95% of extract rich in linoleic acid (65.50%) and α-tocopherol (107.20 mg/kg). The second stage employed supercritical CO_2_ with 15% ethanol–water as a co-solvent to recover medium-polarity phenolics, achieving a total phenolic content (TPC) of 105.35 mg GAE/g DW. Finally, pressurized liquid extraction (PLE) using 58.91% ethanol at 107.98 °C enabled efficient recovery of highly polar fractions, yielding 757.18 mg quercetin equivalents/g of flavonoids, 1508 µg malvidin-3-O-glucoside equivalents/g of anthocyanins, and 258.39 mg epicatechin equivalents/g of condensed tannins.

These advances position SFE as a robust, scalable, and environmentally sustainable alternative to conventional solvent extraction for the recovery of high-value polyphenols from grape by-products.

#### 3.2.6. Accelerated Solvent Extraction (ASE)

The principle of ASE consists of maintaining solvents at liquid state by using high temperatures, above their boiling point and below their critical point, and under high pressure [102]. Under these conditions, the physicochemical properties of the solvent are significantly modified. High pressures (10–20 MPa) used in ASE improve solvent penetration into the matrix pores and keep the solvent in the liquid. Simultaneously, high temperatures (50–200 °C) reduce solvent viscosity and surface tension while enhancing the diffusivity and solubility of polyphenols [103,104]. The main advantage of ASE lies in its short extraction times, higher yields and minimized degradation of polyphenols. Many parameters may affect the efficiency of ASE technology, such as solvent, temperature, pressure, extraction time and number of cycles, and matrix nature [105]. Other than temperature and pressure, solvent choice remains one of the most important parameters to optimize [106]. Hydroethanolic mixtures (ethanol and water) are preferred as green solvents for the recovery of polyphenols from grape pomace due to their ability to extract polar and nonpolar compounds [94,107]. Acidification of the solvent (pH 2) further stabilizes anthocyanins by forming flavylium cations. Optimized ASE conditions (55% ethanol, 141 °C, and 20 min) enabled the efficient recovery of bioactive compounds from grape pomace, resulting in high extraction yields, enhanced total phenolic and proanthocyanidin contents and strong antioxidant activity [107].

ASE has demonstrated superior efficiency in recovering phenolic compounds from grape pomace compared to conventional extraction techniques. Several studies have reported high TPC values in PLE extracts of grape pomace. Pereira et al. obtained TPC values of up to 65.68 mg GAE/g DW using hydroalcoholic solvents at optimized conditions [94]. They also showed that ASE has been highly effective in recovering anthocyanins, with concentrations reaching 10.21 mg/g dry matter under acidic ethanol–water systems.

Recent developments in ASE have focused on enhancing its efficiency and sustainability by coupling it with auxiliary techniques like Enzyme-Assisted Extraction (EAE) or by replacing traditional solvents with NaDES. Enzymes such as cellulase and pectinase catalyze the hydrolysis of structural polysaccharides in the cell wall, facilitating the penetration of the solvent and the release of polyphenols. Machado et al. tested the combination of cellulase treatment prior to ASE for polyphenol extraction from grape pomace [103]. Spectrophotometric analyses showed a decrease in the total phenolic and flavonoid contents, while HPLC analysis demonstrated the positive impact of enzyme treatment on the recovery of specific compounds, especially myricetin and gallic acid. Humanan-Castilla et al. showed that the use of ethylene glycol and glycerol as NaDES in combination with PLE exhibited high recovery of polyphenols from grape pomace [60].

#### 3.2.7. Comparison of Technologies

Table 3 provides a comparative overview of the principal innovative extraction technologies currently applied for the recovery of polyphenols from grape by-products. It highlights key performance indicators, such as extraction efficiency, selectivity, operational advantages and limitations, and level of industrial maturity. A direct quantitative comparison among extraction studies remains challenging because reported efficiencies are strongly influenced by grape variety, by-product composition, pretreatment, extraction solvent, solid-to-liquid ratio and analytical methodology. Moreover, total phenolic assays and chromatographic quantification provide fundamentally different measures of extraction performance.

### 3.3. Fortification of Food Products with Grape Pomace

#### 3.3.1. Bakery Products

Grape pomace (GP) is increasingly recognized as a nutraceutical-rich winery by-product due to its high content of polyphenols, flavonoids, anthocyanins, tocopherols, tocotrienols, dietary fiber, and minerals, making it a promising functional ingredient for cereal-based foods. Bakery and cereal products are particularly suitable carriers because of their high consumption, formulation flexibility, and ability to incorporate GP-derived bioactives while improving nutritional quality and antioxidant capacity [108]. Across breads, biscuits, cookies, muffins, cakes, cereal bars, breadsticks, pizza bases, and pastas, GP incorporation consistently enhances phenolic content, antioxidant activity, dietary fiber, and mineral composition, although excessive substitution may impair technological and sensory quality, as shown in Table 4.

In biscuits and cookies, GP addition markedly improves nutritional and functional properties. Partial substitution of wheat flour with wine lees flour at 10% and 20% enhanced biscuit quality compared to control wheat biscuits, increasing fiber, protein, polyphenols, and antioxidant activity [109]. Theagarajan et al. reported that cookies containing 6% GP showed improved sensory quality, antioxidant activity (IC_50_ of 0.70 mg/mL of extract and total phenolics of 4 mg GAE/g of cookies), reduced anthocyanin degradation, and better hardness retention after 60 days of storage [110]. Giosuè et al. further showed that 20–30% crude GP substitution improved fiber content, lowered fat and caloric value, and supplied up to 5.40 mg GAE/g DW of polyphenols, with high consumer acceptance, especially at 20% [111]. GP also contributes metabolic benefits through fiber- and polyphenol-mediated inhibition of α-amylase and α-glucosidase, potentially slowing carbohydrate digestion and glucose absorption [112]. Antoniolli et al. reported that GP-enriched muffins, biscuits, and cereal bars increased phenolics, ash, protein, anthocyanins, and non-anthocyanin polyphenols while decreasing carbohydrates by 6%, 13%, and 14%, respectively [113]. In addition, GP cereal bars and biscuits provided 3.31 g of fiber 100/g and 5.83 g of fiber/100 g, contributing to the FAO/WHO fiber recommendation of 25–28 g per 2000 kcal.

In bread, GP fortification produces a dose-dependent rise in phenolic content and antioxidant capacity. Sporin et al. reported 5.92 and 3.65 mg GAE/g DW for bread at 15% GP compared with <0.69 mg GAE/g DW in controls [114]. Scappaticci et al. showed that lyophilized red GP combined with pectin increased bread polyphenols without thermal degradation and reduced simple sugars [115]. Tolve et al. demonstrated that 5–10% GP reduced loaf volume and pH but increased phenolics, antioxidant activity, and dietary fiber; at 10%, dietary fiber increased by 150–300%, meeting the EU “high-fiber” claim threshold of >6 g/100 g, while FRAP and ABTS antioxidant capacity increased 3–5-fold [116]. Lactic sourdough-type fermentation of grape pomace-enriched doughs liberates bound phenolic compounds, generates beneficial organic acids, and produces exopolysaccharides that improve dough handling properties [108].

Similar trends have been observed with grape seed powder/flour. Kapcsandi et al. found that 9% grape seed powder increased wheat bread phenolics from 0.91 to 3.16 mg GAE/g DW and antioxidant capacity from 0.70 to 6.44 mg AAE/g; in gluten-free bread, 9% fortification increased polyphenols by 325.90% to 5.92 mg GAE/g DW and antioxidant activity by 294.10% [117]. Oprea et al. showed that 3–5% grape seed flour improved fiber, zinc, and copper while maintaining sensory and rheological quality, whereas 7–9% negatively affected dough behavior, water absorption, crumb structure, color, and consumer acceptance [118]. Elkatry et al. similarly identified 10% grape seed powder as optimal in balady bread, since higher levels reduced dough stability and acceptability [119].

Muffins fortified with GP show increased fiber and polyphenols but greater firmness and lower cohesiveness [120], while smaller particle sizes (212–150 μm) improve anthocyanins, phenolics, and antioxidant activity but darken color and increase hardness [121], while Troilo et al. [122] demonstrated that 15% grape marc flour yielded muffins with high antioxidant activity and >3 g/100 g of dietary fiber, meeting the “source-of-fiber” claim under EC Regulation 1924/2006 [131]. In breadsticks, 5–10% GP increases phenolics, antioxidant capacity, and fiber, enabling “high-fiber” claims, although oxidation and texture changes may occur [123,124]. In pasta, GP flour doubles dietary fiber and isoprenoids and increases soluble polyphenols ten-fold, but excessive inclusion weakens sensory and structural quality; recommended levels range from approximately 4.6% grape peel to 6% GP for egg pasta [125,126,127]. Pizza bases enriched with 15% GP show improved phenolics, antioxidant capacity, and fiber > 6 g/100 g, whereas 25% causes bitterness, acidity, and texture defects [128].

Cereal bars fortified with grape pomace flour exhibited increased water retention and shelf stability due to high pectin content, although texture softness increased [129]. Sensory analysis revealed improved visual appeal despite color changes. Cakes enriched with 4–10% GP exhibit increased phenolics, flavonoids, fiber, and antioxidant activity, rising from 4.10 mg/kg DM in controls to 26.40–60.90 mg/kg DM, with 4% being the most acceptable formulation [10]. Overall, GP is a valuable circular-economy ingredient for functional bakery and cereal products, with optimal incorporation generally depending on product type, particle size, grape fraction, and sensory tolerance.

Lasik-Kurdys et al. demonstrated that jelly candies enriched with dealcoholized winery by-products present a promising approach as they showed higher levels of phenolic compounds and antioxidant levels, although their intense wine-like taste and astringency require optimization to be more consumer-friendly [130].

#### 3.3.2. Dairy Products

Dairy products are among the most promising food matrices for the valorization of grape pomace because they combine high consumer acceptance with physicochemical properties that facilitate the incorporation and stabilization of bioactive compounds. The relatively neutral flavor profile of dairy products, together with their probiotic potential and protein-rich matrix, enables the delivery of phenolic compounds while enhancing the nutritional and functional value of the final product [132]. Given that dairy products account for more than 40% of the functional food market, GP fortification represents an attractive strategy to simultaneously increase dietary polyphenol intake and improve the functional quality of fermented dairy foods [133,134]. Furthermore, interactions between milk proteins, particularly caseins, and grape polyphenols may protect these compounds against oxidation and degradation during storage, although such interactions may also influence their bio-accessibility after digestion.

Among fermented dairy products, yogurt has received the greatest research attention due to its favorable physicochemical environment for preserving polyphenolic compounds and supporting probiotic microorganisms. Yogurt has emerged as the most extensively investigated dairy carrier for GP because its acidic pH (approximately 4.00–4.50) contributes to the stabilization of anthocyanins and other phenolic compounds during storage. Across different studies, GP incorporation consistently increased total phenolic content and antioxidant capacity while generally maintaining or improving microbial viability. Numerous studies consistently demonstrate that incorporating 0.50–5% GP or grape-derived ingredients significantly enhances phenolic content, antioxidant activity, and probiotic survival without adversely affecting the fundamental composition of yogurt [132,135,136,137]. Beyond nutritional improvements, GP also positively influences the technological properties of yogurt. The high dietary fiber content of GP improves water-holding capacity and reduces syneresis, resulting in firmer gels with enhanced consistency and compactness. For instance, yogurts fortified with 2% GP and flaxseed oil exhibited reduced syneresis together with increased acidity, gel strength, phenolic content, and antioxidant capacity [138]. Likewise, combining GP with red cabbage powder stimulated the growth of Lactobacillus delbrueckii ssp. bulgaricus and Streptococcus thermophilus, contributing to improved microbial stability during refrigerated storage [139]. Collectively, these findings indicate that GP acts not only as a source of antioxidants but also as a multifunctional ingredient capable of improving both the nutritional and technological quality of fermented dairy products.

Similar benefits have been reported in cheese, although the response is more dependent on formulation and incorporation levels. Cheese matrices effectively retain grape polyphenols because of strong interactions between phenolic compounds and casein proteins, leading to substantial increases in antioxidant capacity. Cheeses enriched with red and white GP exhibited five- to ten-fold higher phenolic and flavonoid contents than control cheeses, while the addition of only 1% GP powder significantly increased phenolic concentration and enhanced lipoperoxyl radical scavenging activity without adversely affecting fermentation performance or sensory acceptability [140,141]. Likewise, incorporating 5% GP powder into spreadable cheese markedly increased phenolic content, flavonoid concentration, and antioxidant capacity [142]. Nevertheless, several studies indicate that increasing GP concentrations beyond the optimal level may negatively affect rheological properties by increasing hardness and reducing spreadability, highlighting the importance of balancing functional enhancement with consumer acceptability [140,142].

Other dairy products have also been studied. For example, grape skin extracts incorporated into kefir increased TPC by approximately 43%, enhanced overall antioxidant activity by nearly 48%, and significantly reduced DPPH and ABTS IC_50_ values, demonstrating enhanced radical scavenging capacity [143]. Similarly, goat milk fortified with grape extracts exhibited dose-dependent increases in phenolic compounds and antioxidant activity across several complementary assays, including TAC, FRAP, DPPH, and ABTS, confirming the broad antioxidant potential of grape-derived phytochemicals [144].

Overall, the available evidence consistently demonstrates that grape pomace is an effective multifunctional ingredient for dairy products. Across various matrices, GP enhances phenolic content, antioxidant capacity, and, in many cases, probiotic viability while improving technological characteristics, such as water retention and gel structure. However, these benefits are highly dependent on the incorporation level, as excessive GP addition may negatively affect texture, color, and sensory acceptance. Future research should therefore focus on optimizing formulation strategies, elucidating polyphenol–protein interactions and evaluating the bio-accessibility and bioavailability of grape-derived phenolics after gastrointestinal digestion to maximize the health benefits of GP-enriched functional foods.

#### 3.3.3. Meat Products

Meat and fish products are among the most suitable food matrices for grape pomace valorization because they are highly susceptible to oxidative deterioration, owing to their elevated concentrations of unsaturated lipids and pro-oxidant heme compounds. Oxidative degradation not only accelerates rancidity but also compromises color stability, nutritional quality, texture, and shelf life. Consequently, GP has attracted considerable attention as a natural multifunctional ingredient capable of simultaneously providing antioxidant, antimicrobial, and technological benefits while reducing dependence on synthetic preservatives [132]. Across sausages, burgers, patties, and other processed meat products, GP incorporation generally ranges between 0.50 and 3%, consistently improving oxidative stability and reducing malondialdehyde (MDA), a major indicator of secondary lipid oxidation, by approximately 40–70% during refrigerated or frozen storage. These improvements contribute to better preservation of color, flavor, and nutritionally valuable polyunsaturated fatty acids, including omega-3 and omega-6 fatty acids. An overview of representative studies is presented in Table 5.

The antioxidant efficacy of GP has been consistently demonstrated across different meat systems regardless of the incorporation strategy. In pork products, both direct incorporation and edible coating approaches effectively reduced lipid and protein oxidation. Ref. [145] reported that chitosan coatings enriched with grape by-products significantly extended the shelf life of chilled pork through the complementary antioxidant activities of chitosan and grape phenolics. Similarly, direct GP incorporation into pork burgers and sausages effectively limited oxidative deterioration while improving microbiological stability and nutritional quality [146,147,148]. These protective effects are primarily attributed to the ability of grape polyphenols to scavenge reactive oxygen species, chelate transition metals involved in lipid oxidation, and interrupt free-radical chain reactions responsible for quality deterioration.

Beyond oxidative protection, GP also influences the technological and sensory properties of meat products. The high dietary fiber content of GP enhances water- and fat-binding capacities, thereby improving cooking yields, emulsion stability, and product juiciness. However, these benefits are concentration dependent. While supplementation levels of approximately 1–2% generally maintain or even improve consumer acceptance, higher concentrations may increase product firmness, introduce a grainy texture, and negatively affect color attributes [132,149]. In reduced-nitrite beef sausages, for example, 2% GP improved flavor perception but simultaneously decreased lightness, redness, and yellowness. Likewise, the visual impact depends on the grape variety, with red GP imparting darker purple-red hues, whereas white GP or purified extracts provide antioxidant protection with comparatively smaller changes in product appearance [150]. These findings emphasize the importance of optimizing GP concentration to maximize functional benefits without compromising sensory quality.

Comparable improvements have also been observed in poultry and seafood products. Salmon burgers enriched with 1–2% GP exhibited increased dietary fiber and significantly enhanced resistance to lipid oxidation, although the darker coloration reduced consumer acceptance [151]. Similarly, both freeze-dried and microencapsulated GP effectively reduced lipid oxidation in chicken pâté [152], while the incorporation of GP in mechanically deboned chicken meat products maintained acceptable appearance and consumer acceptance at optimized concentrations [153]. These results indicate that the antioxidant properties of GP are effective across different animal protein matrices despite differences in lipid composition and processing conditions.

In addition to its antioxidant capacity, GP possesses considerable antimicrobial potential that further contributes to shelf-life extension. Grape polyphenols exhibit both bacteriostatic and bactericidal activities by disrupting microbial cell membranes, altering membrane permeability, inhibiting essential metabolic enzymes, and interfering with nucleic acid synthesis. These mechanisms have demonstrated inhibitory effects against major foodborne pathogens and spoilage microorganisms, including *Listeria monocytogenes*, *Salmonella* spp., and *Escherichia coli* [154]. Consequently, GP functions as a multifunctional natural preservative capable of simultaneously reducing oxidative deterioration and microbial spoilage, making it a promising clean-label alternative to synthetic antioxidants and antimicrobial additives in meat and fish products.

#### 3.3.4. Other Food Products

Although dairy, bakery and meat products remain the principal focus, GP has also demonstrated considerable potential in other food systems. In chocolate, incorporation levels of 7–10% improved phenolic content, antioxidant properties, and rheological characteristics while simultaneously reducing formulation costs through the partial replacement of conventional ingredients [155]. More recently, Angile et al. reported that replacing cocoa with 5–10% grape pomace flour further enhanced biological activity and total phenolic content, demonstrating the versatility of GP as a functional ingredient beyond dairy applications [156].

The functional potential of GP also extends to plant-based dairy alternatives. In tofu, GP incorporation at 2.50–5% increased hardness and color intensity while producing remarkable improvements in antioxidant capacity (9- to 80-fold) and polyphenol content (2- to 10-fold) compared with conventional formulations [157]. These findings suggest that GP can simultaneously enhance nutritional quality, provide natural pigmentation, and improve oxidative stability in plant-based protein matrices.

Considering the different food applications discussed above, a repeated pattern can be observed between the functional benefits of grape pomace incorporation and the technological and sensory quality of the final products. Increasing GP incorporation can enhance dietary fiber, phenolic content, and antioxidant properties; however, higher addition levels may adversely affect color, texture, rheological behavior, flavor, and overall sensory acceptance. Therefore, no universal upper incorporation level can be established, and the optimal GP concentration should be determined for each food matrix according to the characteristics of the pomace, product formulation, processing conditions, and desired technological and sensory attributes.

### 3.4. Health Benefits of Polyphenols

**Figure 3:**
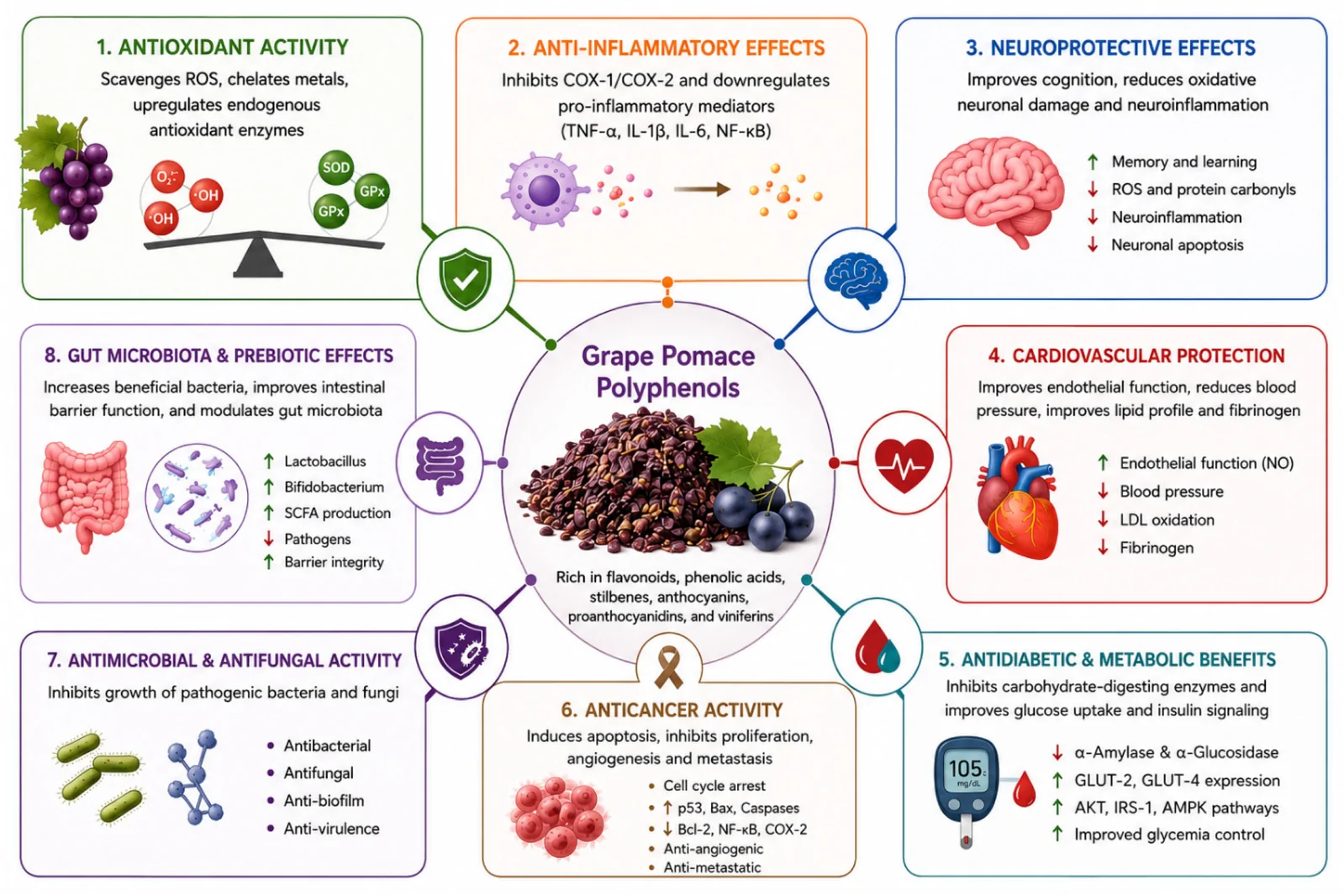
Health-promoting effects of grape pomace polyphenols. Green arrows indicate an increase, whereas red arrows indicate a decrease.

The biological activities of grape pomace polyphenols are summarized in Figure 3, which illustrates their multifunctional health benefits. All the reported health benefits in this section represent a synthesis of findings obtained across different levels of evidence, including in vitro, in vivo, and, where available, human studies.

Extensive evidence from the literature demonstrates that grape-derived polyphenols possess strong antioxidant and anti-inflammatory properties, contributing to the prevention of oxidative stress and chronic non-communicable diseases [44,158]. Their antioxidant activity results from direct radical scavenging, metal ion chelation, and stimulation of endogenous antioxidant enzymes, while anti-inflammatory effects include inhibition of COX-1/COX-2 and suppression of pro-inflammatory mediators [11,159,160,161].

Available evidence suggests that grape polyphenols may have potential neuroprotective, antidiabetic, and cardiovascular effects. Polyphenols improve cognitive performance, reduce oxidative neuronal damage, and suppress inflammatory mediators, such as TNF-α, IL-1β, IL-6, and NF-κB [47,162,163,164,165]. In parallel, they improve glucose metabolism by inhibiting α-amylase and α-glucosidase and activating GLUT-2/GLUT-4, AKT, IRS-1, and AMPK pathways [166,167,168,169]. Cardiovascular protection is mediated through improved endothelial function, enhanced nitric oxide bioavailability, reduced blood pressure, and improved lipid profile and antithrombotic activity [47,158,170,171,172,173,174,175,176,177].

Grape pomace polyphenols also showed anticancer potential. Resveratrol, proanthocyanidins, anthocyanins, and viniferins inhibit cancer cell proliferation, induce apoptosis, and modulate key signaling pathways, including p53, Bcl-2, caspases, and NF-κB, while exhibiting selectivity toward malignant cells [47,158,178,179,180,181,182,183]. These findings support the potential application of grape pomace polyphenols as natural chemopreventive agents.

Finally, grape polyphenols exhibit broad antimicrobial, antifungal, and prebiotic activities. They inhibit both Gram-positive and Gram-negative pathogens, suppress fungal growth, promote probiotic bacteria, and beneficially modulate the gut microbiota by increasing beneficial genera while strengthening intestinal barrier integrity [11,47,184,185,186,187,188,189,190,191]. Collectively, these multifunctional biological activities underscore the considerable potential of grape pomace polyphenols as sustainable bioactive compounds for disease prevention and health promotion.

### 3.5. Agricultural Application: Aerobic Digestion (Composting) and Vermicomposting

The use of organic fertilizers and conventional farming methods has grown increasingly widespread nowadays, showing a stride forward in the circular economy. Since Graefe’s work in 1982, studies on the valorization of grape pomace as a fertilizer have increased, focusing on the heat and CO_2_ emitted [192]. The use of grape pomace as a fertilizer is not recommended due to its long degradation period, possible presence of pathogens and propensity to create catabolites and habiting root growth and seed germination [193]. Grape pomace without any pretreatment applied on wheat induced abiotic stress, although it can be utilized as a bio-pesticide [194]. The benefits could be enhanced by using grape marc compost as a biofertilizer alone or together with other substances. Research on the mixture of grape pomace with other ingredients to be used on grapevine fields resulted in an improvement in fruit quality, crop growth, and soil phosphorus and humus contents [195]. The decision between composting and vermicomposting relies on the scale and the infrastructure and product requirements.

Composting is an aerobic thermophilic bioconversion process of organic waste driven by indigenous microbial consortia under controlled conditions, which results in stability and humification of organic materials. Composting is a well-known, economical and scalable approach in the wine industry for the management and valorization of large volumes of residues, especially grape pomace, by transforming them into agronomically valuable organic fertilizers to be reincorporated into vineyard soils [196]. The high moisture content (around 50–70%), low pH (3.50–4.50) and presence of polyphenolic compounds with antimicrobial activity, which may limit microbial succession and organic matter stabilization, require process control during conventional composting of grape pomace [197]. Co-composting using carbon-rich bulking agents (e.g., straw and wood chips) and nitrogenous substrates (e.g., animal manures) is a typical practice to optimize the carbon-to-nitrogen ratio (preferably 25–30:1), regulate moisture levels, and dilute inhibitory phenolics [198]. Under proper control circumstances, grape pomace can be transformed into mature compost in roughly 60–120 days, producing a stabilized product with a near neutral pH (6.50–7.50), humified organic matter and reduced phytotoxicity.

Composting of grape pomace promotes the degradation of unstable phytotoxic compounds and suppresses pathogenic microorganisms. Earthworms are also capable of partial decomposition of phytotoxic chemicals, hence vermicompost could be an efficient solution and is known to have a lower cost and lesser environmental impact [199]. Vermicomposting consists of using earthworm species, especially Eisenia fetida and Eisenia andrei, to accelerate the biodegradation of organic substrates and to improve the physicochemical and biological quality of the end product [200]. Earthworms mechanically fracture grape pomace, increasing the surface area of the substrate and favoring microbial colonization. Their intestinal and cutaneous secretions also contribute to enzymatic activities that favor the solubilization and transformation of nutrients.

Raw grape pomace has high acidity and a high carbon-to-nitrogen (C/N) ratio, which could limit its direct application in agriculture and have negative impacts on soil and plant systems. Hence, biological stabilization is necessary to turn this waste into a safe and agronomically useful product. Vermicomposting has proven to be a successful and environmentally sustainable approach for the biotransformation of grape pomace into a mature high-quality organic fertilizer.

The conversion of grape pomace into stabilized vermicompost is usually a two-stage bio-oxidative process. The first thermophilic pre-composting phase often lasts 30–40 days and aims to stimulate aerobic decomposition, reduce readily biodegradable substances and achieve good hygiene levels by means of temperatures higher than 50 °C. This is followed by a vermicomposting phase, during which selected earthworm species, most often Eisenia fetida, will further breakdown and convert the substrate over a period of around 90–100 days, permitting organic matter stabilization and earthworm population cycling. Optimal process conditions (moisture of 70–85% and temperatures < 28 °C) are important to maintain earthworm activity and maximum biodegradation efficiency.

Vermicomposting significantly changed the physicochemical and biological properties of grape leftovers. The strong reduction in the C/N ratio, a well-known indicator of compost maturity, indicates a process of nutrient stabilization [201]. However, substrates with high contents of recalcitrant compounds, such as lignin (e.g., vine pruning), may require additional nitrogen-rich amendments to achieve desirable agronomic levels [201]. Meanwhile, the biotransformation mediated by earthworms also contributes to detoxification since the total polyphenol content is greatly reduced, which thus alleviates the antibacterial and phytotoxic effects of fresh grape by-products. The germination index (GI) is a regularly used method to measure the maturity of compost and reduction in phytotoxicity. While vermicompost derived from rotten grape clusters may contain residual salinity, vermicompost based on vine pruning has been found to achieve GI values above 200%, which not only shows the absence of phytotoxicity but also suggests possible biostimulant effects [200].

The vermicomposting process is greatly affected by the initial composition of residues in terms of efficiency and quality. A mixture of grape pomace, vine shoots and wine lees (usually in a 2:1:1 ratio), which are residues of the wine business [202], is a nutritionally balanced substrate for the growth and reproduction of earthworms. Co-vermicomposting of such residues with sewage sludge increases mineral content, including Na, Mg, Zn and Pb; however, the presence of pollutants, especially ammonium, may negatively impair earthworm performance [202]. Also, dealcoholized grape pomace, obtained after industrial recovery of ethanol and pigments, can be successfully vermicomposted to obtain spent seedless grape pomace with improved agronomic value, particularly for use as soil mulch or in the rehabilitation of mining-impacted substrates [29,203,204]. Conversely, vermicompost obtained from rotten grape clusters are generally rich in macro- and micronutrients, especially P, K, S and B, but often have high salinity and residual phytotoxicity that can limit their direct agricultural use without further treatment [205].

The agricultural application of grape pomace-based vermicompost has shown significant benefits in the area of viticulture. At the soil and plant level, vermicompost amendments increase the stable organic matter content and improve nitrogen availability [206,207]. Foliar analyses at the veraison stage show improved uptake of vital nutrients, particularly nitrogen and potassium [202]. These gains are reflected in improved must quality, with increased yeast assimilable nitrogen (YAN) and sugar content, both of which are necessary to guarantee successful alcoholic fermentation [202,206]. Moreover, the employment of organic fertilization systems based on vermicompost has a good effect on wine aromatic profiles, promoting a more complex and stable volatile composition and an increased concentration of esters, higher alcohols and terpenes such as limonene. These modifications collectively lead to better sensory qualities vs. wines made under standard regimes of mineral fertilization [101]. Vermicomposts have higher-quality organic compounds than traditional composts, increasing nutrient bioavailability and improving the capacity to improve soil structure and stimulate microbial abundance and activity in supplemented soils.

### 3.6. Anaerobic Digestion

Anaerobic digestion (AD) represents a strategic and well-established biotechnology for the conversion of renewable organic resources into biogas, particularly the abundant agri-food residues generated by the wine industry [208]. Within a circular bioeconomy framework, AD is increasingly prioritized over less sustainable waste management practices, such as landfilling or incineration, due to its dual capacity to recover bioenergy and mitigate environmental burdens [209]. By valorizing organic residues into a renewable energy carrier, anaerobic digestion contributes to resource efficiency while significantly reducing the ecological footprint of winemaking activities.

Biogas production from grape pomace generally falls within the range of 200–400 L/kg of volatile solids; however, its relatively high lignin content (approximately 15–30%) and elevated concentrations of polyphenolic compounds may inhibit microbial metabolism and reduce methane yields [210]. Although phenolic compounds exhibit valuable antimicrobial properties, they pose significant constraints in anaerobic digestion systems by suppressing methanogenic consortia. To mitigate these limitations, several pretreatment approaches—such as ligninolytic enzymatic hydrolysis, thermal processing, and co-digestion with nitrogen-rich substrates—have been reported to enhance biogas productivity while preserving digestate quality.

Optimizing anaerobic digestion for enhanced biogas productivity requires the integration of advanced process technologies and precise operational control. Among these, anaerobic membrane bioreactors have emerged as one of the most promising innovations, enabling improved biomass retention, higher organic loading rates, and greater process stability [208]. Effective biogas production further depends on real-time monitoring and automated control systems to regulate key operational parameters and maintain stable reactor performance. Accurate physicochemical characterization of feedstocks is also essential to formulate balanced substrate mixtures, thereby improving digester stability and maximizing methane yields [211,212]. In parallel, interdisciplinary advancements, including the application of nanomaterials to stimulate microbial activity and the deployment of artificial neural networks for predictive modeling and process optimization, are increasingly explored [213]. At the microbial level, high-throughput approaches such as metagenomics and proteomics are providing deeper insights into the complex consortia and metabolic pathways governing anaerobic conversion, thereby enabling a more rational process design and targeted performance enhancement as per the same authors.

The wine industry generates a wide spectrum of biodegradable residues suitable for anaerobic digestion, including grape pomace, seeds, stems, and wine lees. Within integrated biorefinery schemes, solid fractions remaining after supercritical CO_2_ extraction of high-value compounds can be further valorized through anaerobic digestion, reinforcing the cascading use of biomass [214]. Additionally, grapes and winery by-products are employed in vinegar production through sequential anaerobic and aerobic fermentations, highlighting the versatility of grape-derived substrates within bioprocessing chains [215].

From an industrial and logistical perspective, the implementation of anaerobic digestion is most effective when integrated into on-site or regional bioprocessing platforms, where the generated biogas can be directly utilized to offset local energy demands. Given the economic and technical limitations associated with biogas transport, localized consumption is generally preferred [208]. However, small- and medium-sized wineries often face seasonal variability in residue availability, which can compromise digester performance. This challenge can be alleviated through cooperative schemes among multiple producers to ensure a consistent and diversified supply of organic substrates [211,212].

Beyond its technological relevance, anaerobic digestion plays a critical environmental role by supporting the transition toward low-carbon energy systems. Approximately 20–25% of the grape harvest is converted into solid and semi-solid residues during winemaking, representing a substantial organic waste stream. The controlled anaerobic valorization of these by-products prevents their uncontrolled degradation, reduces greenhouse gas emissions associated with landfilling, and provides a renewable alternative to fossil-based energy carriers. Consequently, anaerobic digestion constitutes a pivotal component in sustainable winery management and circular bioresource utilization [216].

### 3.7. Application in Wine Industry

The increasing adoption of circular economy principles in the wine sector has stimulated the valorization of grape and winery by-products as multifunctional oenological ingredients. Extracts rich in polysaccharides, polyphenols, tannins, and grape cell wall materials have emerged as sustainable alternatives to conventional additives, offering applications in wine clarification, stabilization, aroma preservation, sensory enhancement, microbial control, and reducing Sulphur dioxide (SO_2_) usage [217,218,219,220,221].

The effects of these extracts depend largely on their origin, purification level, and molecular composition. Grape-derived extracts are rich in pectic polysaccharides, including rhamnogalacturonan II (RG-II) and polysaccharides rich in arabinose and galactose (PRAG), whereas yeast-derived products mainly contain mannoproteins and β-glucans. Their addition generally increases wine polysaccharide content while modifying phenolic composition through adsorption mechanisms. Although grape pomace- and must-derived extracts have only minor effects on total phenolics, purified wine polysaccharides and yeast derivatives reduce tannins and hydroxybenzoic acids, contributing to lower astringency and improved mouthfeel [217]. Similarly, purified grape pomace exhibits a strong affinity for condensed tannins and has been proposed as a sustainable fining agent comparable to bentonite, PVPP, and isinglass while simultaneously supporting cleaner-label and vegan-friendly wine production [221,222].

Grape- and wine-derived polysaccharides also influence wine aroma through interactions with volatile compounds. Canalejo et al. demonstrated that polysaccharide extracts recovered from grape pomace, grape must, and wine lees enhanced the concentration of fruity and floral volatile compounds during bottle ageing, particularly when rich in mannoproteins, glucans, non-pectic polysaccharides, and low-molecular-weight fractions [219]. In contrast, purified grape pomace used as a fining agent caused only slight reductions in esters and terpenes while preserving the majority of volatile compounds, producing effects comparable to commercial fining agents [222]. These findings highlight the potential of winery by-products to simultaneously improve aroma stability and contribute to sustainable wine production.

Beyond aroma, grape polysaccharides positively influence wine sensory properties. RG-II and PRAG reduce bitterness and astringency through interactions with phenolic compounds and salivary proteins while enhancing sweetness perception, whereas mannoprotein-rich preparations improve body, roundness, and palate persistence [223,224,225]. However, contradictory results regarding bitterness, acidity, and sweetness have been reported, suggesting that these effects depend on wine composition, polysaccharide structure, and dosage [217,226]. Aroma expression is also influenced by extract origin, with grape-derived fractions enhancing fruity notes and yeast-derived products promoting lees-related sensory attributes [217].

From a technological perspective, mannoproteins improve protein and tartaric stability, RG-II regulates tartrate crystallization, and PRAG-rich fractions influence filtration performance [226,227]. Furthermore, polyphenolic extracts recovered from grape by-products exhibit antimicrobial activity against *Brettanomyces bruxellensis* and *Acetobacter pasteurianus*, reducing spoilage and offering the possibility of lowering SO_2_ additions [228]. Nevertheless, current evidence indicates that these extracts should be considered complementary rather than complete substitutes for Sulphur dioxide.

Despite their considerable potential, several limitations remain. Valle et al. reported that polyphenolic extracts caused undesirable browning in white wines, probably due to co-extracted pigmented phenolics, which may negatively affect consumer acceptance [228]. In addition, the chemical composition and technological performance of grape by-product extracts vary according to grape variety, extraction method, and degree of purification [28]. Consequently, further research is required to optimize extraction protocols and application strategies that maximize functional benefits while preserving wine color, aroma, and overall sensory quality.

Overall, grape and winery by-product extracts represent promising multifunctional oenological ingredients capable of improving wine quality while supporting sustainable winery management and circular economy approaches. Their successful industrial implementation will depend on standardized extraction processes and a better understanding of the molecular interactions governing their effects on wine composition and sensory properties.

### 3.8. Animal Feed

Winemaking by-products are increasingly recognized as valuable functional feed ingredients that improve animal health, productivity, and the quality of animal-derived products. Rich in polyphenols, dietary fiber, and unsaturated fatty acids, grape pomace and related extracts help counteract oxidative stress and inflammation, thereby enhancing the oxidative stability of meat, milk, and eggs while supporting more sustainable livestock production [229]. Red grape pomace, particularly fractions rich in grape pulp, also provides nutritional benefits by improving feed digestibility and contributing to better meat and milk quality through its high polyphenol and linoleic acid content [230,231].

In pigs, grape polyphenols have shown promising antioxidant and anti-inflammatory effects, particularly during the stressful post-weaning period. Supplementation with 5% grape pomace increased feed intake without impairing growth while enhancing the activity of antioxidant enzymes, including catalase, glutathione peroxidase, and superoxide dismutase [229]. Although growth responses have varied among studies, dietary grape pomace or grape extracts generally improved growth performance and reduced intestinal inflammation by modulating gut microbiota, enhancing digestive enzyme secretion, and improving nutrient utilization [57,232,233,234].

Grape by-products also improve the quality and oxidative stability of animal products. In poultry, low dietary inclusion levels (<3%)—particularly when grape pomace was biologically fermented—enhanced growth performance and antioxidant capacity and reduced pathogenic bacteria, such as *Clostridium perfringens* [235]. Dietary grape by-products or grape seed extracts reduced lipid oxidation in broiler meat, increased tissue tocopherol concentrations, improved water-holding capacity, and enhanced meat nutritional quality [234,236]. In laying hens, grape by-products also increased egg weight [237]. Similarly, supplementation in sheep improved redox balance, reproductive performance, and meat tenderness while reducing lipid peroxidation [229,238].

In dairy production, grape pomace supplementation has been associated with improvements in milk functionality. Diets containing 15% dried grape pomace increased lactose and lactoglobulin concentrations, while grape polyphenols transferred into milk enhanced antioxidant activity, reduced mammary inflammation, and improved shelf life [234]. However, responses are not always consistent, as no significant changes in milk composition were observed in Friesian cows receiving 10% grape by-products [239]. Ensiling technologies, including bale silage, further improve the practical use of grape pomace by preserving its nutritional and bioactive properties for up to 180 days, enabling year-round utilization in ruminant feeding systems [240].

Despite these advantages, excessive dietary inclusion of grape by-products may reduce nutrient digestibility because of their high tannin, anthocyanin, and lignified fiber contents, and high concentrations of secondary metabolites may adversely affect animal performance in sensitive species [230,241,242]. Overall, the efficacy of grape polyphenols depends on their chemical composition, bioavailability, dosage, and the physiological status of the animal. When appropriately incorporated into livestock diets, grape by-products improve the antioxidant potential and nutritional quality of meat, milk, and eggs while extending shelf life through reduced lipid peroxidation [243]. Nevertheless, optimized species-specific feeding strategies remain necessary to maximize their benefits while minimizing potential adverse effects.

### 3.9. Cosmetic Applications

Grape pomace and its derived extracts have gained considerable attention in the cosmetic and personal care industries due to their well-documented antioxidant, anti-aging, anti-inflammatory, and photoprotective properties [244]. The skin health-promoting potential of grape polyphenols closely aligns with current consumer demand for natural, sustainable, and multifunctional cosmetic ingredients. By mitigating damage induced by ultraviolet radiation, atmospheric pollutants, and environmental stressors, such as wind, humidity, and temperature fluctuations, grape-derived polyphenols contribute to the preservation of the skin lipid barrier and the structural integrity of cellular proteins [245].

Red grape pomace extracts incorporated into lipid-based formulations have demonstrated remarkable broad-spectrum photoprotective activity in reconstructed human epidermis models [246]. Treatment with these extracts significantly reduced UV-induced production of reactive oxygen species (ROS), nitric oxide (NO), and interleukin-1α (IL-1α) by approximately 60–65%, while maintaining cell viability above 90%. Histological evaluations further confirmed epidermal structural preservation, characterized by reduced dyskeratosis, restored intercellular cohesion, and normalized keratinization following combined UVA/UVB exposure.

Extensive investigations have characterized the polyphenolic profiles of grape pomace extracts and assessed their suitability as bioactive cosmetic ingredients. Major compounds of interest include catechin, epicatechin, gallic acid, and resveratrol, in addition to associated fatty acids and vitamins. These constituents exert anti-aging effects primarily through inhibition of collagenase and elastase, key proteolytic enzymes responsible for dermal extracellular matrix degradation. Notably, grape pomace extracts obtained using natural deep eutectic solvents exhibited significant inhibition of collagenase-mediated gelatin degradation and promoted enhanced cell migration in scratch assays, supporting their wound-healing potential and cosmetic anti-aging efficacy [247]. Wittenauer et al. further reported that low-molecular-weight hydrophilic polyphenols, particularly gallic acid, were the most effective enzyme inhibitors and confirmed the strong radical-scavenging capacity of grape by-product polyphenols [248].

Beyond pomace, grape stem extracts have demonstrated significant depigmenting activity while maintaining excellent safety profiles in keratinocyte and fibroblast models [249]. The combined antioxidant, anti-inflammatory, and tyrosinase-inhibitory properties highlight grape by-products as promising multifunctional cosmetic agents.

To overcome limitations related to polyphenol stability, bioavailability, and skin penetration, nano-formulation and microencapsulation strategies are increasingly being explored [250]. These delivery systems protect sensitive compounds from oxidative degradation and improve the dermal transport of hydrophobic bioactive compounds, such as resveratrol, thereby enhancing their efficacy in cosmetic applications.

### 3.10. Additional Applications

#### 3.10.1. Packaging

The development of biodegradable polymers derived from agricultural and food processing wastes has attracted increasing scientific interest as a sustainable alternative to conventional plastic materials, which are major contributors to environmental pollution [251]. Anthocyanins, present in high concentrations in grape pomace, possess significant functional value and have therefore been widely investigated for incorporation into polymeric films as bioactive and intelligent packaging materials [252,253]. Intelligent packaging systems are designed to interact with food matrices through the release and adsorption of specific compounds, thereby providing a visible indication of product quality and freshness [254]. Anthocyanins exhibit distinct color transitions—from red to purple and dark blue—in response to pH changes ranging from acidic to alkaline conditions. Such pH variations are associated with chemical, enzymatic, and microbiological processes, including the production of volatile amines and organic acids, which serve as indicators of food spoilage. Nogueira et al. demonstrated that bioactive packaging films containing 40% grape pomace extract exhibited a pronounced color change from red to green during fish spoilage, confirming the effectiveness of grape pomace anthocyanins as visual sensors for monitoring food freshness [251]. The incorporation of grape pomace extracts into chitosan films reinforces mechanical properties while providing excellent antioxidant activity. The films incorporated with extracts exhibited enhanced water resilience compared to unmodified chitosan, improving performance under high-humidity conditions typical in food packaging [255].

#### 3.10.2. Biofuel

Projections toward 2050 indicate a substantial increase in global energy demand driven by population growth, necessitating the transition toward more sustainable and diversified energy systems [256,257]. In parallel, research into bioenergy has intensified as part of global efforts to mitigate greenhouse gas emissions and reduce dependence on fossil fuels. Grape-processing by-products represent a promising and underexploited biomass resource for bioenergy generation, as their individual components contribute distinctly to various biofuel pathways. The high cellulose and hemicellulose contents of grape pomace can be hydrolyzed into fermentable sugars for bioethanol production, whereas lignin-rich fractions are suitable precursors for bio-oil generation through pyrolytic conversion. Moreover, the abundant polyphenolic compounds, including tannins, anthocyanins, and flavonoids, provide intrinsic antioxidant protection during biomass storage, thereby improving feedstock stability and preserving its suitability for downstream biofuel production [258]. Nevertheless, the high lignin content necessitates energy-intensive and technologically demanding pretreatment processes, which remain a key bottleneck in efficient bioconversion. In addition, variability in pomace composition arising from differences in grape varieties and winemaking practices leads to heterogeneous bioenergy yields. Despite these constraints, the multiple conversion pathways and renewable nature of grape pomace underscore its significant potential as a sustainable bioenergy resource [258].

Thermochemical conversion of grape by-products at temperatures exceeding 800 °C through gasification has been shown to generate economically and environmentally viable syngas streams suitable for hydrogen production [259]. Alternatively, François et al. demonstrated that grape pomace could serve both as a fermentation substrate and as a source of endogenous microbial consortia for biological hydrogen production [260]. Anaerobic digestion of grape pomace offers another valorization route, producing methane-rich biogas alongside a solid digestate that can be recycled as an agricultural fertilizer. Mechanical pretreatments, particularly grinding, have been reported to significantly enhance biogas yields when applied prior to anaerobic digestion [261].

In addition to gaseous biofuels, grape by-products can be exploited for liquid biofuel production. Transesterification of fatty acids extracted from grape seeds yields fatty acid methyl esters, which exhibit superior fuel properties and stability compared with ethyl esters, highlighting their suitability for biodiesel applications [262]. Thermochemical processes such as combustion, gasification, and pyrolysis convert grape pomace into energy-rich gas, biochar, and liquid fractions, which can subsequently be upgraded into diesel- and gasoline-range fuels via catalytic pathways [263]. Biochemical conversion routes have also demonstrated high efficiency. Corbin et al. reported ethanol yields of approximately 400 L per ton of grape pomace following pretreatment and saccharification [264], while Mendes et al. obtained about 310 L per ton of grape skins, confirming the strong potential of winery residues for bioethanol production [265].

Beyond energy generation, recent research has explored the integration of grape by-products into bio-based composite materials. Ref. [266] evaluated grape seeds, wine lees, and grape pomace as bio-fillers in poly (butylene adipate-co-terephthalate) (PBAT) and polybutylene succinate (PBS) matrices. At filler loadings of 30–40%, the resulting composites maintained thermal stability while exhibiting significantly enhanced stiffness, supporting the broader role of winery by-products in sustainable, circular bioeconomy frameworks.

#### 3.10.3. Kombucha

The growing prevalence of chronic diseases and increasing consumer awareness of the relationship between diet and health have driven a rapid expansion of the functional beverage market. Among these products, beverages enriched with bioactive compounds have attracted considerable scientific and industrial interest because they combine health-promoting properties with high consumer acceptance, convenience, and favorable shelf stability [267]. This trend is particularly pronounced in the Middle East and Africa, where the non-alcoholic beverage sector has experienced increasing demand for fermented functional drinks, with kombucha emerging as one of the fastest-growing products [268].

Kombucha is a traditional fermented beverage originating from East Asia approximately 2000 years ago, where it was first consumed in China and later spread to Japan and other regions. It is produced by fermenting sweetened tea with a symbiotic culture of bacteria and yeasts (SCOBY), consisting primarily of acetic acid bacteria, lactic acid bacteria, and various yeast species [269,270]. During fermentation, microbial metabolism generates a wide range of organic acids, polyphenol-derived metabolites, vitamins, and other bioactive compounds that contribute to kombucha’s characteristic sweet–sour flavor and its reported biological activities, including antioxidant, antimicrobial, anti-inflammatory, hepatoprotective, anti-hypercholesterolemic, and anticancer effects [271].

Recently, the use of grape and winery by-products as fermentation substrates has emerged as an innovative strategy that combines agro-industrial waste valorization with the production of next-generation functional beverages. The fermentation of grape pomace by kombucha microorganisms enables the biotransformation of this polyphenol-rich by-product into beverages with enhanced nutritional, sensory, and functional properties while contributing to circular economy objectives [2]. Owing to the enzymatic activity of the SCOBY consortium, complex phenolic compounds are released and transformed into more bioavailable metabolites, thereby increasing the biological value of the final beverage. For example, Andayani et al. reported that grape supplementation during kombucha fermentation significantly enhanced the antioxidant capacity of the beverage [272]. Likewise, Barakat et al. demonstrated that kombucha fermentation of grape pomace increased total polyphenol content by approximately 140%, together with higher anthocyanin concentrations, resulting in a 50–75% improvement in antioxidant, anti-inflammatory, and antidiabetic activities [2]. These findings highlight kombucha fermentation as a promising biotechnological platform for the sustainable valorization of grape pomace, enabling the development of innovative, value-added functional beverages with enhanced health-promoting potential.

#### 3.10.4. Other Applications

Currently, the majority of grape-processing by-products are valorized as soil amendments and organic fertilizers due to their high content of organic matter and essential nutrients [273]. However, the intensive and continuous application of these residues has contributed to nutrient oversaturation in soils, resulting in physicochemical imbalances, reduced soil fertility, and an increased risk of environmental contamination [274]. Consequently, alternative and higher value-added valorization strategies are being explored, including the distillation of fermented grape pomace for the production of pomace spirits. These beverages are obtained through the distillation of fermented pomace, allowing for the recovery of volatile aromatic compounds and the production of high-alcohol-content spirits. Pomace spirits are traditionally consumed as digestifs, exemplified by Italian grappa, and are increasingly incorporated into alcoholic cocktails. In addition, they are employed in fortified winemaking, such as Port wine production, where they are used to arrest fermentation by increasing ethanol concentration [263]. Beyond beverage applications, pomace spirits also serve as culinary ingredients, contributing aromatic complexity to sauces and desserts. The non-volatile fractions and solid residues remaining after distillation are further repurposed into various agricultural and industrial applications, reinforcing the circular economy potential of winery by-products. In parallel, winery by-products have attracted growing interest as sustainable sources of natural colorants. Recent studies have demonstrated their applicability as biodyes for textile materials, including cotton, cationized cotton, and wool, offering an ecofriendly alternative to synthetic dyes [275]. Visco et al. investigated the development of disposable cutlery formulated from grape flour blended with other plant-based flours, incorporating xanthan gum and palm oil, as a sustainable alternative to conventional petroleum-based plastics [276]. An emerging and sustainable approach involves the valorization of grape by-products to produce efficient sorbents aimed at mitigating heavy-metal contamination in wastewater. Microporous activated charbon derived from grape pomace has demonstrated a strong capacity to adsorb Mn (VII) from industrial effluents [277] and Pb^2+^ from wastewater systems [278]. In parallel, grape-based biochar has gained increasing attention as a multifunctional material, serving not only as a substrate for bioenergy production [279] but also as a cost-effective alternative to activated carbon, exhibiting similar performance [93].

## 4. Conclusions and Perspectives

The transition of the wine industry toward a circular bioeconomy requires viewing winery by-products not as waste streams but as valuable renewable resources. Grape pomace, seeds, skins, stems, wine lees, and winery wastewater constitute abundant reservoirs of polyphenols, dietary fibers, lipids, proteins, and other bioactive compounds that can be converted into high-value products for the food, nutraceutical, cosmetic, pharmaceutical, agricultural, and environmental sectors. Their successful valorization simultaneously addresses environmental concerns associated with waste disposal while creating new economic opportunities for wineries and associated industries.

Recent advances in extraction technologies have significantly improved the recovery of these valuable compounds. Although conventional solvent extraction remains the most widely implemented approach, emerging green technologies—including ultrasound-assisted extraction, microwave-assisted extraction, pulsed electric fields, high-voltage electrical discharges, accelerated solvent extraction, and supercritical fluid extraction—offer improved extraction efficiency with reduced solvent consumption, shorter processing times, and lower environmental impacts. Increasingly, the combination of complementary technologies, together with green solvents, such as natural deep eutectic solvents, represents one of the most promising strategies for maximizing extraction yields while preserving the biological activity of sensitive compounds. From an industrial implementation perspective, UAE and PEF appear among the more readily scalable technologies, supported by their increasing application in food-processing systems. In contrast, the broader implementation of HVED and MAE remains constrained by scale-up and equipment-related challenges, while SFE and ASE technologies, despite their industrial potential, may be limited by specialized equipment requirements and relatively high capital and operating costs.

Beyond compound recovery, winery by-products have demonstrated remarkable versatility in diverse applications ranging from functional food ingredients and natural antioxidants to edible packaging materials, animal feed, biofuels, fermented beverages, natural colorants, biosorbents, and biodegradable materials. Such multifunctional applications reinforce the concept of integrated biorefineries, where multiple value-added products can be generated from the same biomass, thereby improving overall process profitability and sustainability.

Despite these advances, several challenges remain before widespread industrial implementation can be achieved. The heterogeneous composition of winery residues, influenced by grape variety, climatic conditions, viticultural practices, and vinification processes, complicates process standardization and quality control. Furthermore, the scalability of emerging extraction technologies, their techno-economic viability, energy requirements, and regulatory compliance require further investigation. Standardized analytical methodologies and harmonized quality criteria for winery-derived extracts are also needed to facilitate industrial adoption and market acceptance.

Future research should therefore prioritize the integration of green extraction technologies with digital process optimization, artificial intelligence, process intensification, and continuous biorefinery systems to improve efficiency and reduce production costs. Greater emphasis should also be placed on comprehensive life-cycle assessment, techno-economic analysis, and carbon footprint evaluation to objectively quantify the environmental benefits of winery by-product valorization. In parallel, advances in encapsulation technologies, controlled-release systems, and precision formulation will further expand the application of winery-derived bioactive compounds in functional foods, personalized nutrition, and biomedical applications. Finally, stronger collaboration among researchers, industry, policymakers, and regulatory agencies will be essential to establish harmonized legislation and accelerate technology transfer from laboratory-scale research to industrial production.

Overall, the sustainable valorization of winery by-products represents a major opportunity to simultaneously enhance resource efficiency, reduce environmental burdens, and generate innovative bio-based products. Continued technological innovation and multidisciplinary research will be instrumental in transforming the wine industry into a more resilient, competitive, and sustainable sector while contributing to global circular economy objectives.

## Figures and Tables

**Table 1:** Chemical composition, influencing factors, and functional applications of major grape and winery by-products.

By-Product	Major Chemical Classes	Notable Compounds	Influencing Factors	Functional Properties	Applications
Grape Pomace	Polyphenols, dietary fibers, proteins, pectin, fatty acids, and vitamins	Anthocyanins (red), flavan-3-ols (white), catechins, procyanidins, galacturonic acid, linoleic acid, and tocopherols	Grape variety, vinification, extraction method, and climate	Antioxidant and dietary fiber	Food additive, nutraceutical, and cosmetics
Grape Seeds	Polyphenols, fatty acids, tocopherols, and proteins	Catechin, epicatechin, gallic acid, linoleic acid, and γ-tocopherol	Variety, region, and processing	Antioxidant and source of unsaturated lipids	Nutraceuticals, edible oil, and cosmetics
Grape Stems	Polyphenols (proanthocyanidins and stilbenes) and dietary fibers	(−)-Epicatechin polymers, syringic acid, resveratrol, and cellulose	Variety, altitude, soil, and extraction	Antioxidant and anti-aging	Food ingredients, nutraceuticals, and cosmetics
Wine Lees	Polyphenols, proteins, and polysaccharides	Mannoproteins, arabinogalactans, and anthocyanins	Fermentation, yeast strain, and aging	Emulsifier and antioxidant	Food ingredients, nutraceuticals, and cosmetics

**Table 2:** Solid–liquid extraction of different pomaces with different parameters.

Grape Pomace Type	Operating Parameters	Targeted Analyses	Reference
Red and white grapes	4g in 100 mL of solvent for 2 h at 50 °C with agitation	Total polyphenols, flavanols, and anthocyanins	[50]
Agiorgitiko grape	Drying; water, water: ethanol (1:1) and ethanol; 3g in 100 mL of solvent; 2 to 6 h	Total polyphenols, anthocyanins, flavonoids, flavonols and antioxidant capacity	[51]
Red grape	Solvent: ethanol/water, 80%; 20 g in 100 mL with agitation	Total phenolics and antioxidant activity	[52]
Merlot skins and seeds	A total of 0.3 g in 20 mL of 50% aqueous ethanol; acidity: 1 N of HCl (0, 0.5 and 1%); 30, 45 and 60 min, all at 80 °C	Total phenolics, hydroxycinnamic acids and antioxidant activity	[53]
Red grape	Water and ethanol (8–92%); solid–liquid ratio of 1:3 and 1:17; 1 h at 30 °C with agitation	Total phenolics, anthocyanins, phenolic acids, flavonols and antioxidant activity	[54]
Cabernet Sauvignon pomace	A total of 5 g in 100 mL of solvent, ethanol, water, and water/ethanol (3:1, 1:1, and 1:3), and ethyl acetate at 25 °C, 35 °C and 45 °C	Total phenolic compounds, total flavonoid content, total flavonols and antioxidant activity	[55]
Red grape	Ratio of 1:20). Water and ethanol (30/70) at 50 °C for 1 h, followed by 24 h extraction at room temperature. Water (100%) at 70 °C and 100 °C for 1 h, followed by 24 h extraction at room temperature. Water (100%) at room temperature for 24 h.	Total polyphenol content, total tannins, total anthocyanin content, antioxidant activity, anti-inflammatory activity, and polyphenol profiling	[56]
Touriga Nacional and Sousão pomace	Solvent of 50%, 70%, and 90% ethanol; temperature of 20 °C, 40 °C, and 60 °C; and pH of 0.5% HCl, 2% HCl, and 3.5% HCl	Total phenolics and flavonoids, radical scavenging capacity, and polyphenol profiling	[36]
Red and white grapes	A total of 40 mg of extract in 1.5 mL of solvent, with ethanol:water (0:100, 25:75, 50:50, 75:25, and 100:0), for 30 min with orbital shaking	Total phenolics and flavonoids, ortho-diphenol content, antioxidant activity, and polyphenol profiling	[57]

**Table 3:** Comparative assessment of emerging extraction technologies for polyphenol recovery.

Technique	Main Advantages	Main Disadvantages	Polyphenol Efficiency	Selectivity	Scalability/ Industrial Readiness *
**PEF**	Non-thermal; preserves bioactives; low energy; enhances mass transfer; reduces solvent use	Not a standalone extraction; requires solvent step; high equipment cost; dependent on matrix conductivity	Moderate (as standalone); high when combined with other extraction processes	Low (acts as pretreatment)	Industrial as pretreatment
**HVED**	Strong cell disruption; rapid extraction; no bulk heating	Radical formation may degrade phenolics; high energy consumption; electrode erosion; complex control	High but variable (risk of degradation)	Low–moderate	Pilot scale
**UAE**	Low cost; simple; scalable; reduced solvent/time; mild conditions	Limited penetration at large scale; possible degradation at high intensity; moderate selectivity	High (especially for total phenolics)	Moderate	Widely industrialized
**MAE**	Very fast; high yields; efficient for polar compounds; low solvent use	Thermal degradation risk; non-uniform heating; scale-up challenges	High (fast extraction)	Moderate–high (for polar compounds)	Semi-industrial
**SFE**	Solvent-free extracts; high selectivity; environmentally friendly; no solvent residues	Low efficiency for polar phenolics without co-solvent; high capital cost; complex optimization	Moderate–high (with co-solvent)	High	Industrialized
**ASE**	High yields; short extraction time; reduced solvent use; compatible with green solvents; automated	Risk of thermal degradation; equipment cost; mostly batch systems	Very high (for polar and semi-polar phenolics)	Moderate–high	Growing industrial use

* Industrial maturity represents a qualitative assessment based on the implementation scale reported in the reviewed literature rather than a formal TRL classification. “Pilot scale” indicates evidence of operation beyond laboratory scale; “semi-industrial” indicates scale-up or demonstration under conditions approaching industrial operation; and “industrialized” indicates documented industrial or commercial implementation. Categories indicating “growing industrial use” refer to technologies for which industrial applications have been reported but whose implementation remains less widespread.

**Table 4:** Applications of grape pomace extracts in fortified bakery products.

Extract Used	Product Fortified	Effects	Reference
Grape pomace	Bakery and cereal products	Increased phenolic content, antioxidant activity, dietary fiber, minerals, and overall nutritional quality; excessive levels may reduce technological and sensory quality.	[108]
Wine lees flour, 10% and 20%	Biscuits	Improved biscuit quality; increased fiber, protein, polyphenols, and antioxidant activity compared with control wheat biscuits.	[109]
Grape pomace, 6%	Cookies	Improved sensory quality and antioxidant activity; IC_50_ = 0.70 mg/mL of extract; total phenolics = 4 mg GAE/g of cookies; reduced anthocyanin degradation and improved hardness retention after 60 days.	[110]
Crude grape pomace, 20–30%	Bakery products/cookies	Increased fiber content, lowered fat and caloric value, and supplied up to 540 mg GAE/100 g of polyphenols; high consumer acceptance, especially at 20%.	[111]
Grape pomace	Cereal-based foods	Potential inhibition of α-amylase and α-glucosidase, possibly slowing carbohydrate digestion and glucose absorption.	[112]
Grape pomace	Muffins, biscuits, and cereal bars	Increased phenolics, ash, protein, anthocyanins, and non-anthocyanin polyphenols; decreased carbohydrates by 6%, 13%, and 14%, respectively.	[113]
Grape pomace	Cereal bars and biscuits	Improved fiber content; cereal bars contained 3.31 g of fiber/100 g and biscuits contained 5.83 g of fiber/100 g.	[113]
Grape pomace, 15%	Bread	Increased phenolic content to 5.92 and 3.65 mg GAE/g DW compared with <0.69 mg GAE/g in controls.	[114]
Lyophilized red grape pomace with pectin	Bread	Increased bread polyphenols without thermal degradation and reduced simple sugars.	[115]
Grape pomace, 5–10%	Bread	Reduced loaf volume and pH but increased phenolics, antioxidant activity, and dietary fiber; 10% GP increased dietary fiber by 150–300% and FRAP/ABTS capacity by 3–5-fold.	[116]
Fermented grape pomace-enriched dough	Bread/dough systems	Released bound phenolics, generated organic acids, and produced exopolysaccharides that improved dough handling.	[108]
Grape seed powder, 9%	Wheat bread and gluten-free bread	In wheat bread, phenolics increased 3-fold and antioxidant capacity 9-fold. In gluten-free bread, polyphenols increased by 325.90% and antioxidant activity by 294.10%.	[117]
Grape seed flour, 3–5%	Bread	Improved fiber, zinc, and copper while maintaining sensory and rheological quality; 7–9% negatively affected dough behavior, water absorption, crumb, color, and acceptance.	[118]
Grape seed powder, 10%	Balady bread	Identified as the optimal level; higher levels reduced dough stability and acceptability.	[119]
Grape pomace	Muffins	Increased fiber and polyphenols but increased firmness and reduced cohesiveness.	[120]
Fine grape pomace particles, 212–150 μm	Muffins	Improved anthocyanins, phenolics, and antioxidant activity but darkened color and increased hardness.	[121]
Grape marc flour, 15%	Muffins	Produced muffins with high antioxidant activity and >3 g/100 g of dietary fiber, meeting the “source-of-fiber” claim.	[122]
Grape pomace, 5–10%	Breadsticks	Increased phenolics, antioxidant capacity, and fiber; enabled “high-fiber” claims, although oxidation and texture changes may occur.	[123,124]
Grape pomace flour/grape peel, approx. 4.60–6%	Pasta/egg pasta	Doubled dietary fiber and isoprenoids and increased soluble polyphenols ten-fold; excessive inclusion weakened sensory and structural quality.	[125,126,127]
Grape pomace, 15% and 25%	Pizza bases	A total of 15% improved phenolics, antioxidant capacity, and fiber > 6 g/100 g; 25% caused bitterness, acidity, and texture defects.	[128]
Grape pomace flour	Cereal bars	Increased water retention and shelf stability due to high pectin content; increased softness; improved visual appeal despite color changes.	[129]
Grape pomace, 4–10%	Cakes	Increased phenolics, flavonoids, fiber, and antioxidant activity from 4.1 mg/kg DM in controls to 26.40–60.90 mg/kg DM; 4% was the most acceptable formulation.	[10]
Dealcoholized wine by-products	Jelly candies	Increased phenolic compounds and antioxidant levels; intense wine-like taste and astringency require optimization for consumer acceptance.	[130]

**Table 5:** Valorization of grape pomace in meat food products.

Extract Used	Product Fortified	Effects	Reference
Grape pomace/grape by-products	Meat and fish products	Provided antioxidant, antimicrobial, and nutritional benefits; helped limit lipid oxidation, microbial spoilage, discoloration, and quality loss during storage.	[132]
Grape pomace, 0.50–3%	Sausages, patties, burgers, and processed meats	Improved oxidative stability; reduced malondialdehyde (MDA) formation by 40–70% during refrigerated and frozen storage; helped preserve color, flavor, and omega-3 and omega-6 fatty acids.	[132]
Grape by-products incorporated into chitosan-based coatings	Chilled pork	Extended chilled pork shelf life; phenolics and chitosan acted synergistically to reduce lipid and protein oxidation.	[145]
Grape pomace	Pork burgers	Improved preservation by limiting protein and lipid oxidation.	[146]
Grape pomace	Pork sausages	Enhanced oxidative stability and improved microbiological and nutritional quality.	[147,148]
Grape pomace, 1% and 2%	Beef sausages	Improved consumer acceptance, especially flavor, in a trained panel of 20 assessors; however, in reduced-nitrite formulations, 2% GP decreased lightness, redness, and yellowness.	[149]
Grape pomace fiber	Meat products	Improved water and fat binding, juiciness, and cooking yield; excessive incorporation above 3% may cause undesirable firmness and graininess.	[132]
Red grape pomace/white pomace/decolorized extracts	Meat products	Red GP may produce purple-red tones; white pomace or decolorized extracts provide antioxidant effects with less visual impact.	[150]
Grape pomace, 1% and 2%	Salmon burgers	Increased dietary fiber and strongly protected against lipid oxidation; darker color reduced consumer acceptance.	[151]
Freeze-dried and microencapsulated grape pomace extracts	Chicken pâté	Reduced lipid oxidation compared with the control.	[152]
Grape pomace, up to 120 mg/kg, combined with mechanically deboned chicken meat, up to 15 g/100 g	Chicken nuggets	Did not negatively affect appearance or consumer acceptance.	[153]
Grape polyphenols	Meat and fish products	Showed bacteriostatic and bactericidal activity against spoilage and pathogenic microorganisms, including *Listeria monocytogenes*, *Salmonella* spp., and *Escherichia coli*.	[154]

## Data Availability

No new data were created or analyzed in this study. Data sharing is not applicable to this article.
